# Nanopore sequencing of drug-resistance-associated genes in malaria parasites, *Plasmodium falciparum*

**DOI:** 10.1038/s41598-018-26334-3

**Published:** 2018-05-29

**Authors:** Lucky R. Runtuwene, Josef S. B. Tuda, Arthur E. Mongan, Wojciech Makalowski, Martin C. Frith, Mallika Imwong, Suttipat Srisutham, Lan Anh Nguyen Thi, Nghia Nguyen Tuan, Yuki Eshita, Ryuichiro Maeda, Junya Yamagishi, Yutaka Suzuki

**Affiliations:** 1 Department of Computational Biology and Medical Sciences, Graduate School of Frontier Sciences, The University of Tokyo, 5-1-5 Kashiwanoha, Kashiwa, Chiba 277-8562 Japan; 2 Faculty of Medicine, Sam Ratulangi University, Kampus Unsrat, Bahu Manado 95115 Indonesia; 3 Institute of Bioinformatics, Faculty of Medicine, University of Münster, Niels-Stensen Strasse 14, Münster, 48149 Germany; 4 Artificial Intelligence Research Center, Advanced Industrial Science and Technology, 2-3-26, Aomi, Koto-ku, Tokyo, 135-0064 Japan; 5 AIST-Waseda CBBD-OIL, 3-4-1 Ookubo, Shinjuku-ku, Tokyo, 169-8555 Japan; 6 Department of Molecular Tropical Medicine and Genetics, Faculty of Tropical Medicine, Mahidol University, 420/6 Ratchawithi Road, Thung Phaya Thai, Ratchathewi, Bangkok 10400 Thailand; 7 National Institute of Hygiene and Epidemiology, 1 Yersin Street, Hanoi, 112800 Vietnam; 8 Division of Collaboration and Education, Research Center for Zoonosis Control, Hokkaido University, North 20, West 10 Kita-ku, Sapporo, Hokkaido 001-0020 Japan; 9 Department of Medical Entomology, Faculty of Tropical Medicine, Mahidol University, 420/6 Ratchawithi Road, Thung Phaya Thai, Ratchathewi, Bangkok 10400 Thailand; 10 Division of Biomedical Science, Department of Basic Veterinary Medicine, Obihiro University of Agriculture and Veterinary Medicine, Nishi 2 Sen-11 Inadacho, Obihiro, Hokkaido 080-0834 Japan; 11 Global Station for Zoonosis Control, GI-CoRE, Hokkaido University, North 20, West 10 Kita-ku, Sapporo, Hokkaido 001-0020 Japan

## Abstract

Here, we report the application of a portable sequencer, MinION, for genotyping the malaria parasite *Plasmodium falciparum*. In the present study, an amplicon mixture of nine representative genes causing resistance to anti-malaria drugs is diagnosed. First, we developed the procedure for four laboratory strains (3D7, Dd2, 7G8, and K1), and then applied the developed procedure to ten clinical samples. We sequenced and re-sequenced the samples using the obsolete flow cell R7.3 and the most recent flow cell R9.4. Although the average base-call accuracy of the MinION sequencer was 74.3%, performing >50 reads at a given position improves the accuracy of the SNP call, yielding a precision and recall rate of 0.92 and 0.8, respectively, with flow cell R7.3. These numbers increased significantly with flow cell R9.4, in which the precision and recall are 1 and 0.97, respectively. Based on the SNP information, the drug resistance status in ten clinical samples was inferred. We also analyzed *K13* gene mutations from 54 additional clinical samples as a proof of concept. We found that a novel amino-acid changing variation is dominant in this area. In addition, we performed a small population-based analysis using 3 and 5 cases (*K13*) and 10 and 5 cases (*PfCRT*) from Thailand and Vietnam, respectively. We identified distinct genotypes from the respective regions. This approach will change the standard methodology for the sequencing diagnosis of malaria parasites, especially in developing countries.

## Introduction

Malaria is at the forefront of the WHO’s disease eradication programs^1^. Every year, malaria causes the most fatalities of any parasitic disease, and the most severe symptoms have been reported from *Plasmodium falciparum* infections^2^. Spread all over tropical countries across the world, malaria imposes a severe problem in developing countries. While no vaccine is available for the malaria parasite, anti-malaria drugs are readily available. Indeed, medicines against malaria parasites have been known probably since malaria itself was noted approximately 4,000 years ago^3^. Starting from quinine and progressing to chloroquine, sulfadoxine-pyrimethamine, mefloquine, and the recent artemisinin, these drugs were effective in killing parasites^4^. However, rapidly increasing cases of drug-resistant malaria are gradually rendering medication difficult. Due to administration of drugs at suboptimal concentrations, partly because of inadequate dosing and the half-life of the medicines themselves, parasite strains that are resistant to these medicines started to appear and are rapidly spreading^5^. Particularly, *P. falciparum* has developed resistance to nearly all anti-malarial drugs that are in current use^5,6^. For example, chloroquine-resistant *P. falciparum* has been described everywhere in Central and South America, Africa, and Southeast Asia^7^. Sulfadoxine-pyrimethamine is currently not effective in Africa, Southeast and South Asia, South America, or Oceania^8^. Notably, the most powerful anti-malaria drug, artemisinin, has been shown to have reduced efficacy in Southeast Asia^9^. Recently, it has become known that multidrug-resistant malaria is starting to spread from the Mekong region^10^. To address these concerns, novel anti-malaria drugs are being developed^11^. However, antimalarial invention takes a long time; artemisinin was found in 1972 but its approval as an antimalarial was in the 1990s^12^. There is a pressing need to precisely understand the prevalence of drug resistance and decide the proper strategy for how to use the limited repertoire of drugs and their combinations.

Drug resistance is acquired due to mutations in the parasite genes. There have been several parasite genes reported to be associated with drug resistance. For instance, cytochrome B mutations cause resistance to atovaquone^13^. Mutations in *PfCRT* and *PfMDR1* cause resistance to quinine, chloroquine, amodiaquine, mefloquine, piperaquine, lumefantrine, and primaquine^14^. Mutations *in PfDHFR* and *PfDHPS* cause resistance to sulfadoxine-pyrimethamine^8^. Likewise, mutations in *K13* have been associated with decreased artemisinin susceptibility^15^. To be more precise, those drug-resistant mutations are mostly realized by single nucleotide polymorphisms (SNPs) and their combinations in these genes. For example, mutations in transmembrane domains 1, 4 and 9 of *PfCRT* are responsible for the chloroquine-resistant phenotype^16^. Similarly, four point mutations in *PfDHFR* and four point mutations in *PfDHPS* are known to be responsible for hampering the drug effect of sulfadoxine-pyrimethamine^8^. Five recently described point mutations in the *K13* gene are purported to cause artemisinin resistance^15^. When they occur, *K13* mutations neutralize the drug effect of impairing the PI3K signal pathway in the parasite^17,18^.

To ensure proper use of the drugs, examination of parasites’ genotypes at the early stage of the infections is ideal, preferably before drug administration. For this purpose, sequencing is the most decisive means. Although conclusive, sequencing technologies, including Sanger and massively parallel sequencing, are rarely available in the field hospitals of developing countries, where the immediate diagnoses are needed. A recently available new type of sequencer, MinION, is changing the methodology of sequencing. This technology has a great advantage in addressing the current concerns regarding the use of sequencing technology in developing countries. It does not require a laborious sample processing step by skillful laboratory technicians. Indeed, MinION is a single-use USB-powered sequencer, operated by a PC, requiring no prior instrumental investment^19^. The application of MinION in the field can greatly assist the identification of pathogens, such as Ebola and Zika viruses^20,21^. MinION also has been used to assemble bacterial genome *de novo*^22^. A pathogen’s relatively small genome size means that whole genome sequence can be easily performed using MinION’s current chemistry. Whole genome sequence of *Neisseria gonorrhoae* in clinical setting has helped with the antibiotic selection^23^. Therefore, utilization of MinION is preferable when faced with malaria parasites. The parasite is quick to acquire drug resistance^24^. Drug resistance against artemisinin is recognized after failure to clear malaria parasites in the third day post-medication based on microscopic examination^15^. Sequencing of the respective parasite can predict that resistance is expected and therefore a change of drug regimen might be considered. The data is also important for surveillance or further research in finding out the mechanism of resistance.

Here, we report our first use of the MinION sequencer for genotyping *P. falciparum* in the field.

## Results and Discussion

### Sequencing of laboratory strains

We selected nine genes in *P. falciparum*, namely, mitochondrial apocytochrome B (*CYTB*), sarcoplasmic/endoplasmic reticulum Ca^2+^-ATPase6 (*PfATPase6*), multidrug resistance protein 1 (*PfMRP1*), dihydrofolate reductase-thymidylate synthase (*PfDHFR*), translationally controlled tumor protein (*TCTP*), chloroquine resistance transporter (*PfCRT*), multidrug resistance protein 1 (*PfMDR1*), dihydropteroate synthase (*PfDHPS*), and Kelch protein gene (*K13*), which are associated with resistance to the most representative anti-malaria drugs as shown in Supplementary Table 1. For these genes, we amplified the entire genic regions by PCR and subjected them to MinION sequencing. These genes were not long and had relatively short introns if any. One or two sets of PCR amplicons approximately 1–3 kb long could cover the entire coding regions.

**Figure 1:**
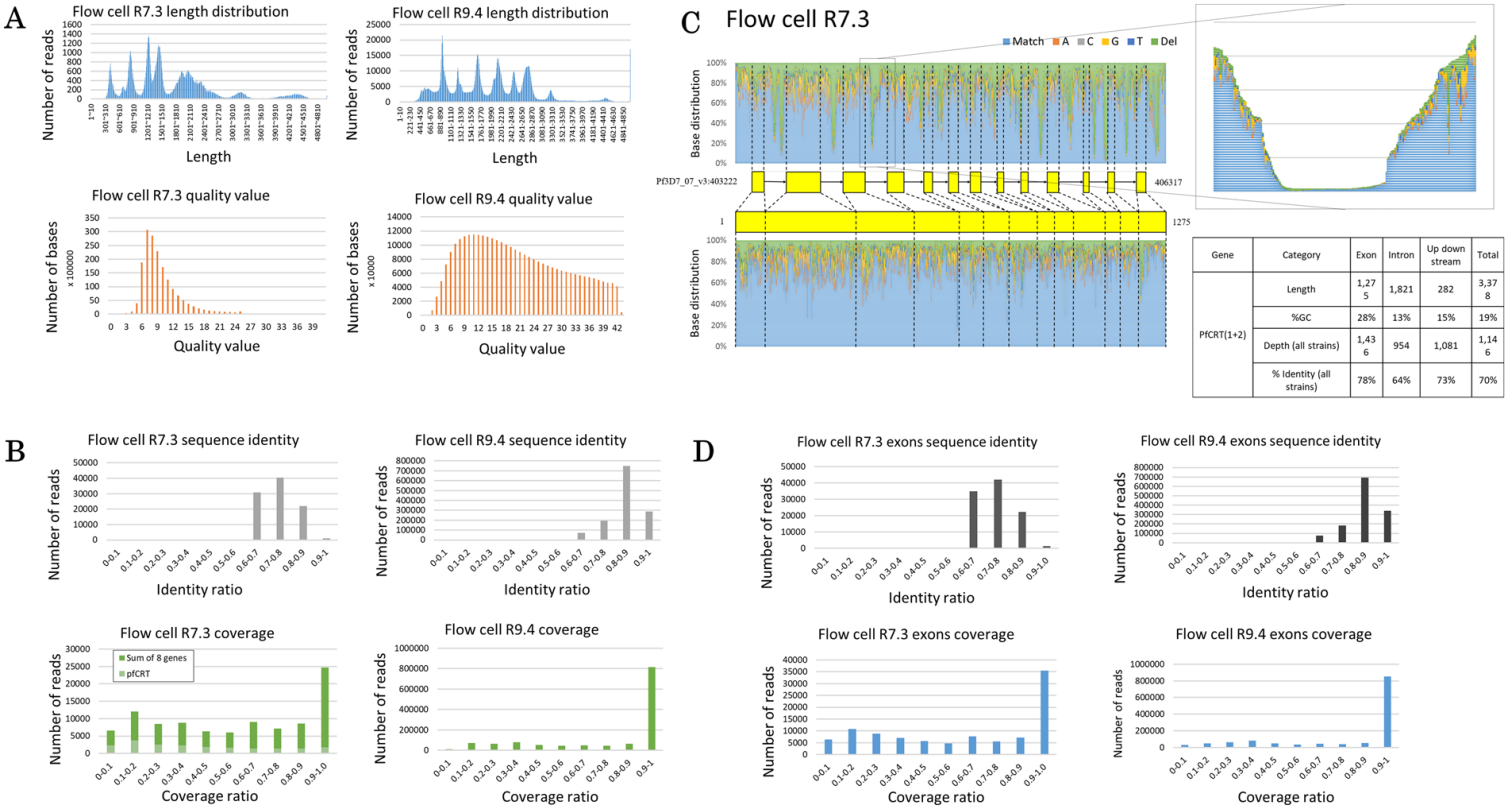
Sequencing statistics of laboratory strains. Nanopore sequencing covers the whole length of nine genes of the laboratory strains regardless of the flow cell version. The quality value is significantly better for flow cell R9.4 (**A**). The sequence accuracy is better for flow cell R9.4. Mapped reads are fragmented with flow cell R7.3, especially *PfCRT* (**B**). The fragmentation is caused by introns (*PfCRT* is shown as an example) having higher AT counts than the exons (lower right panel). Upper left panel shows the SNPs distribution of *PfCRT*, while lower left panel shows the SNPs distribution of *PfCRT* protein-coding regions only. Light blue color symbolizes the matched sequenced nucleotides to the reference genome, while orange, grey, yellow, dark blue, and green colors symbolize the mutation to adenosine, cytosine, guanine, thymine, and deletion, respectively. Upper right panel shows the magnification of the deleted section of a *PfCRT* intron. Data for this figure is obtained with flow cell R7.3 (**C**). When introns are excluded from the analysis, fragmentations decrease with no difference in sequence accuracy on flow cell R7.3, but such problem is not found on flow cell R9.4, even if introns are included in the analysis (**D**). Note the significant increase in read number on flow cell R9.4 in Figures A, B, and D.

We first sequenced the four commonly used laboratory strains, i.e., 3D7, 7G8, K1, and Dd2. The PCR amplicons of the nine genes were sequenced as a mixture, with one MinION flow cell used for each strain. We sequenced the samples using the obsolete flow cell R7.3 and re-sequenced them with the newest flow cell R9.4. We obtained 179,444 reads for the combined laboratory strains with flow cells R7.3 (Supplementary Table 2**)** and 1,875,850 with flow cells R9.4 (Table 1). Their average lengths were 1,654 and 2,736 bp for flow cell R7.3 and R9.4, respectively (Fig. 1A, top panel). MinION’s ability to sequence relatively long reads comes with the drawback of low sequence quality is only true in the obsolete flow cells. The average sequencing quality value (QV) with flow cell R7.3 was 9.4, which increased to 20.1 when re-sequenced with the newest chemistry (Fig. 1A, bottom panel). The general low quality of flow cell R7.3, may be partly caused by the fact that *P. falciparum* is especially difficult organism to sequence. Particularly in the intergenic regions and introns, the AT content exceeded 80%, and there are numerous AT-rich repetitive sequences^25^. Even in the exonic regions, there are several such AT-rich regions. The MinION principle of deciphering the electric disturbance pattern for every five nucleotides in MinION may have made the base-calling of such regions particularly difficult. On the other hand, the newest chemistry upgrades the nanopores to have better accuracy^26^. It also employs new base-calling algorithms based on Recurrent Neural Nets (RNN)^27^. These upgrades might be compatible with the complex malaria parasite genome.

**Table 1:** Sequencing and mapping statistics for four laboratory strains of *P. falciparum*.

Sample	Total reads (2D)	Reads that are mapped to target (LAST 658, MC Frith *et al*. 2010 BMC Bioinformatics)	Ratio of mapped reads
3D7	619,172	605,491	97.79%
K1	222,110	217,971	98.13%
7G8	797,373	754,223	94.59%
Dd2	327,195	318,180	97.24%
Total	1,875,850	1,734,486	92.46%

These results were obtained with flow cell R9.4.

We aligned the obtained reads to the respective genic regions of the reference genome sequence, which is determined based on 3D7 strain. For mapping, we used the alignment program LAST^28^. We tried various parameters and thresholds as shown in Supplementary Figure 1. Although BWA-MEM outperforms LAST in this test, we have opted to use LAST as the mapping software since we are using a match/mismatch score matrix (“ATMAP”) that is theoretically tuned for the AT-richness of this genome^29^; in theory this should improve accuracy. Definitively judging which is the best aligner is difficult. But our paper does not depend on this; it requires only that our alignments are good enough to justify our results and conclusions. With the tuned parameters, 57.86% of reads sequenced with flow cell R7.3 were aligned to the target region (Supplementary Table 2) with 73.46% similarity to the reference (Fig. 1B, top left panel). This is comparable to the sequencing of the AT-rich bacterium FSC996, which also yielded an average 79% read accuracy^30^. Using the new chemistry, however, these numbers greatly increased to 92.46% mapping ratio (Table 1) and 84.56% identity (Fig. 1B, top right panel). During the mapping, we found that the alignments were generally fragmented in the *PfCRT* gene when sequenced with R7.3 (Fig. 1B, bottom left panel). For this gene, most of the alignment blocks cover only 20% of the target region. A similar observation was also obtained in the other amplicons to varying extents (Supplementary Figure 2A). We examined the cause of the fragmentation. For *PfCRT*, we found that the sequence depth was especially low in the introns (Fig. 1C). The introns had an extremely low GC content, and the sequences consisted of series of homopolymers, which imposed serious problems for MinION^31^. When we removed the introns and included exons only for the analysis, the fragmentation problem disappeared (Fig. 1D, bottom left panel). The overall sequencing accuracy also improved to 74.07% (Fig. 1D, top left panel). This problem, however, was not found in flow cell R9.4 as no fragmentations occurred (Fig. 1B, bottom right panel). Excluding introns from the downstream analysis did not generate much difference on the sequence accuracy, either **(**Fig. 1D, right panel).

We examined the possible cause of the mapping errors and found that the incorrectly amplified PCR amplicons were occasionally sequenced preferentially. The primers that we used to finalize sequencing have been chosen from multiple trial-and-errors. We chose the ones that showed the expected amplicon length. Nevertheless, although we confirmed the presence of single band amplifications before the sequencing, those dubious sequences may have been enriched, perhaps due to preferential sequence efficacy. For example, we found that the earlier peaks observed in the *K13* amplicons were derived from incorrectly primed amplicon products (Supplementary Figure 2B). A sequence in the central of the gene has high similarity to the reverse primer, which we postulate can contribute to the mis-priming and give rise to shorter fragment amplification. Furthermore, we do not think this seemingly mis-priming can be solved by alignment alone. When attempts are made to use large sets of sequencing data to reduce the cost per sample, additional care should be taken to further optimize the PCR primers.

### Detection of SNPs

Compiling the obtained sequence reads, we attempted to determine the genotype with respect to each parasite gene of interest. For this purpose, we developed a bioinformatics analytical pipeline. To identify SNPs, we first constructed consensus sequences from MinION reads, following the scheme shown in Supplementary Figure 3. Briefly, the consensus base is detected when the depth at a given position is more than a threshold R, where R is depth variable, and when the ratio of a base to the sum of all bases at a given position is larger than another threshold X. For heterozygous sites, a second base was called in a similar way. When the total read count is less than the threshold R or when no base fulfills the condition defined by the threshold X, the position is considered unknown, “n”. A SNP is called if the consensus base is different from the reference.

**Figure 2:**
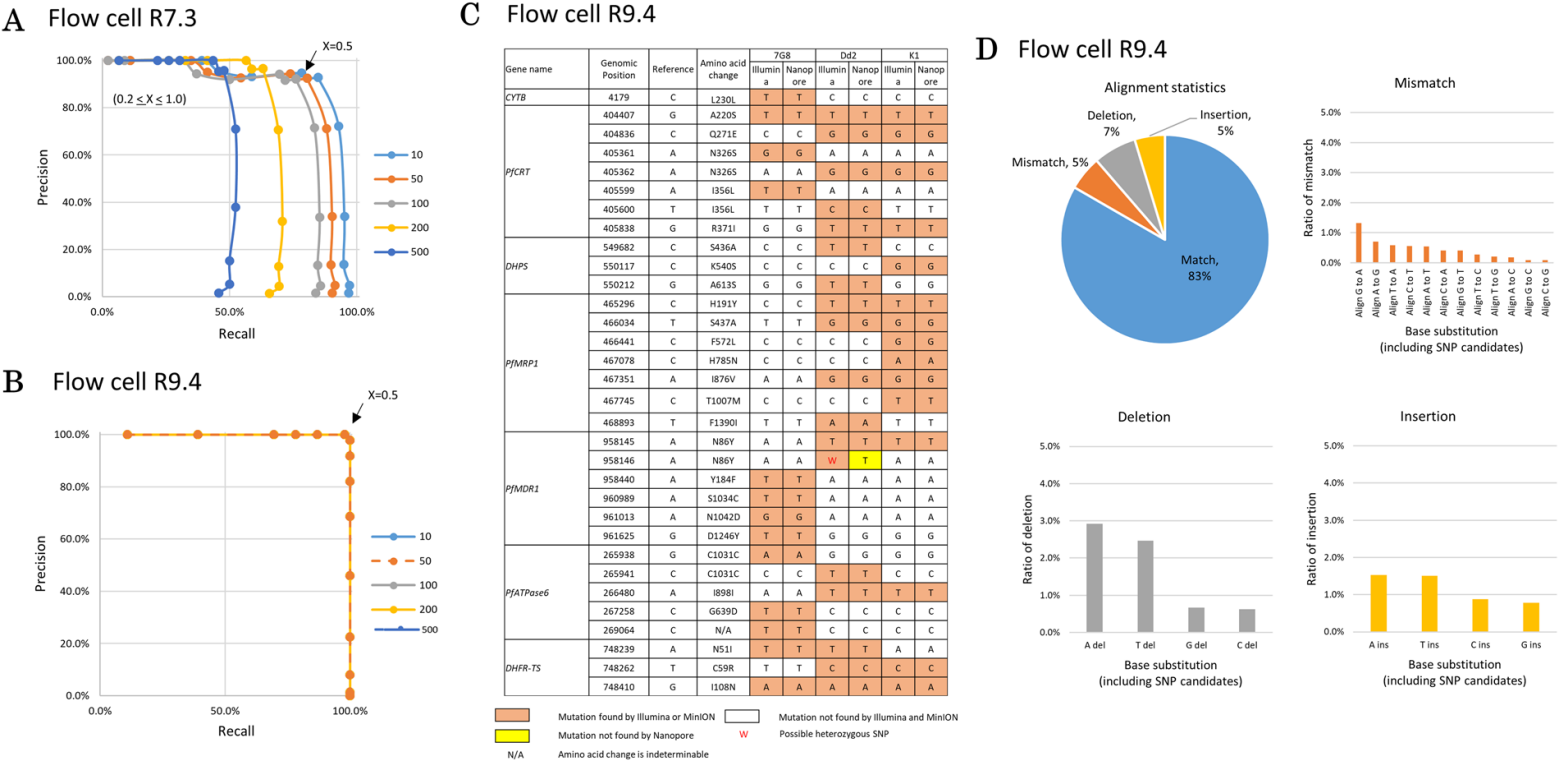
SNP calling of laboratory strains. The precision and recall of multiple thresholds used for heterozygous SNP calling on data obtained with flow cell R7.3. Precision is defined as the ratio of true positive (i.e., SNPs found by both MinION and Illumina) to the total of true positive and false positive (i.e., SNPs found only by MinION). Recall is defined as the ratio of true positive to the total of true positive and false negative (i.e., SNPs found only by Illumina). The lines show the sequence depth used for analysis (threshold R). The dots on the line show the threshold X. Threshold X ≥ 0.5 with R ≥ 50 sequence depth is used for subsequent analysis because of the reasonable precision and recall values, which are 0.92 and 0.8, respectively (**A**). On the other hand, flow cell R9.4 gives the same precision and recall for every threshold R with X ≥ 0.5, which is 1 and 0.97, respectively. In this figure, all the lines produced by different threshold R stack up (**B**). SNPs called with the parameters X ≥ 0.5 and R ≥ 50 on data obtained with flow cell R9.4 show 100% identity to Illumina validation (orange boxes). Exception is made for one position in *PfMDR1* (red color font). This position is seemingly heterozygous as called by Illumina. However, MinION could only determine this position as a homozygous SNP (**C**). The error patterns show that G to A mismatches, A deletions, and A/T insertions are the most frequent polymorphisms in the laboratory strain malaria parasites sequenced with flow cell R9.4 (**D**).

To test the performance of the developed pipeline, we compared the results of the detected SNPs with those from the Illumina sequencing. For Illumina sequencing, the PCR amplicons were subjected to shotgun sequencing. SNPs were called using a standard program, GATK. From the sequencing, we detected 52 SNPs. Using these SNPs as the reference dataset, we evaluated the precision and recall rates of the constructed pipeline at varying parameters of the thresholds R and X. Our analysis revealed that at threshold of R > 50, a reasonable good precision-recall curve was obtained (Fig. 2A). In this curve, threshold of X > 0.5 yielded satisfactorily high precision and recall rates of 0.92 and 0.8, respectively. This was true only for flow cell R7.3, because flow cell R9.4 gave consistent precision and recall with every R value, which are 1 and 0.97, respectively (Fig. 2B). We distinguish SNPs from PCR errors by following the algorithm in Supplementary Figure 3. Since PCR errors are supposed to occur randomly on the elongation of DNA strand, we believe that it would not exceed the threshold X ≥ 0.5 that we employ in our pipeline. An SNP, on the other hand, would exceed the threshold since the SNP should be present in every DNA molecule that is amplified by PCR.

Sequencing with flow cell R7.3, however, showed some false positive and negative calls (Supplementary Figure 4), assuming the calls by the Illumina sequencing are correct. Of the three “false” positive cases, two cases are in either AT-rich regions or homopolymer tracts, which is a challenge for MinION sequencing (see example in Supplementary Figure 5A). Nine “false” negative cases were derived from the “unconfident” call due to the requirements of X or R values being unsatisfied. One interesting case was found in the *PfMDR1* region. In this case, one SNP was heterozygous, which was also confirmed by Sanger sequencing (Supplementary Figure 5B). This heterozygous SNP, which might have been acquired in a minor subpopulation of the parasites before or after the laboratory strain was established, could not be detected due to the unsatisfied requirement of the X parameter. Importantly, all those problems were once common to Illumina sequencing in its early days and have been addressed by specializing protocols for the analysis of *P. falciparum* later. Supplementary Figure 5C shows another example of validating correct SNPs. As expected, data obtained from flow cell R9.4 showed a very different result. While the mutations found with R7.3 were also found with R9.4, our pipeline performed a lot better with the new flow cell. Previously undetermined positions were confidently called. False positive and negative nucleotides also disappeared. Nevertheless, our pipeline could not call the seemingly heterozygous position of *PfMDR1* (Fig. 2C).

For future development, we inspected the causes of incorrect SNP calls. We collected basic information on the sequencing errors in general. We evaluated the error rates occurring in all of the sequence reads sequenced with R9.4 and found that those respective mutation types accounted for 5%, 7% and 5% of the mismatch, deletion, and insertion errors, respectively (Fig. 2D). This is an improvement over flow cell R7.3 (Supplementary Figure 6). Guanine was more likely to be miscalled as adenosine. Deletions were more likely to be accumulated at adenosine or thymine sites. Insertions were mainly adenosine or thymine. Incorrectly called SNPs at the relaxed threshold somewhat represented these general error patterns. Thus, considering the general patterns of the error matrices should give useful information to minimize erroneous SNP calling, especially when relaxed thresholds are used.

To compare the performance of our pipeline, we also employed a variant calling specifically built for MinION, Nanopolish^32^, that uses hidden Markov model (HMM) signal-level consensus algorithm to call for SNPs. As flow cell R7.3 employs HMM for its base-calling, Nanopolish applies the same algorithm for variant-calling. Using default parameters, we found that Nanopolish variant-calling returned many false positive results for the data obtained with R7.3 (Supplementary Figure 7). Even calling the reference strain yielded false positive nucleotides (Additional File 1). There were instances when Nanopolish found some variants not detected by our pipeline, but the many false positive results indicate that Nanopolish might be too sensitive for variant calling, at least in malaria parasite. On the contrary, our pipeline has been optimized for malaria parasite’s genome to minimize false nucleotides.

### Sequencing of clinical samples

**Figure 3:**
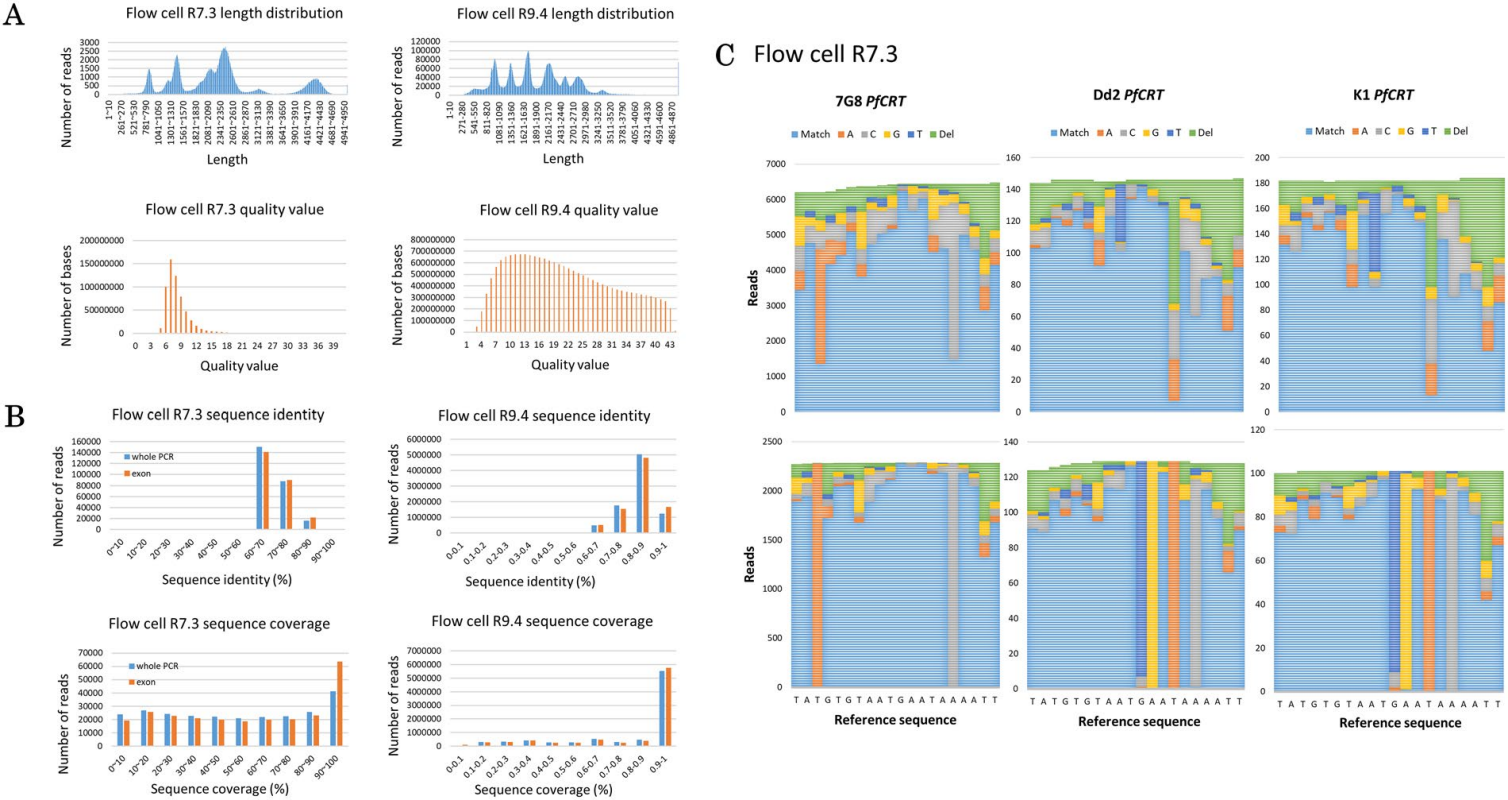
Sequencing of clinical samples. The read length distributions of the combined clinical specimens show that the sequenced reads are distributed to cover all nine genes with a quality value that is significantly better on flow cell R9.4 (**A**). The reads obtained with flow cell R7.3 are not fragmented in clinical specimens, although exons are included in the analysis. Nevertheless, removal of introns significantly increased the coverage. On the other hand, the reads obtained with flow cell R9.4 have better accuracy and coverage, regardless of the introns removal (**B**). Remapping of the sequenced reads (upper panel) to the representative haplotype reference shows confident SNP calling (lower panel). In this process, we removed the reads that have unconfident SNPs called in highly difficult region of amino acid 72–76 of *PfCRT*. We then remapped the remaining reads to the representative strain reference to increase call confidence (**C**). Note the significant increase in read number on flow cell R9.4 in Figures A and B. Comparison between data produced by flow cell R7.3 and R9.4 uses different samples due to the exhaustion of the original samples for the flow cell R7.3 analysis.

We applied the developed technique to analyze clinical samples. First, ten samples from patients with a positive diagnosis of *P. falciparum* infection were processed in the same manner as the laboratory strains. A total of 337,104 reads were obtained for the nine genes of ten clinical samples (Table 2). As the case with laboratory strains, the numbers increased greatly with flow cell R9.4 (Supplementary Table 3). Unfortunately, comparison of flow cell R7.3 and R9.4 performance could not be completed with the same clinical samples, because we have used all of the original samples for the analysis with flow cell R7.3. The average read length of 2,334 bp (Fig. 3A, top left panel) and QV of 8.3 was similar to that of the laboratory strains (Fig. 3A, bottom left panel). On the contrary, sequencing with flow cell R9.4 yielded reads with an average read length of 15.9 kB and QV of 20, which was a very significant improvement (Fig. 3A, right panel). Sequencing accuracy and coverage of data obtained with flow cell R9.4 did not change much with the exclusion of introns, while data from flow cell R7.3 gave a significant improvement in coverage with the exclusion of introns (Fig. 3B).

**Table 2:** Sequencing and mapping statistics of ten clinical samples.

Sample	Total reads (2D)	Mapped reads	Best score (≥150)	Ratio of mapped reads
2-#2	60,472	72,353	40,339	66.7%
2-#5	116,582	64,713	41,273	55.5%
5-#1	34,186	31,468	18,928	92.05%
5-#3	2,493	2,920	1,562	62.65%
5-#6	48,600	49,856	24,701	49.54%
5-#39	2,024	1,807	1,139	89.28%
5-#40	26,663	16,477	9,872	61.8%
5-#41	10,037	8,671	6,901	86.39%
5-#43	19,618	7,892	4,989	40.23%
5-#46	18,922	16,466	12,664	87.02%

These results were obtained with flow cell R7.3.

Using the analytical pipeline described above, we were able to detect SNPs for *PfCRT* and all other genes using data obtained from flow cell R7.4 (Supplementary Table 4). Some SNPs were shared with the laboratory strains, while novel candidates were also identified uniquely in clinical samples and had never been documented before. There were instances when MinION gave false positive results. As with laboratory strains, these false positive cases were not in the coding region, indicating the difficulty of MinION to sequence the intronic region. Furthermore, the high number of false positives in only one sample raised the suspicion of poor library preparation that caused relatively poor MinION sequencing. Overall, MinION’s precision and recall for this dataset are 0.87 and 0.91, respectively.

In addition to the SNPs occurring at a single base, there is one site in the *PfCRT* gene where different haplotypes were directly related to chloroquine resistance. The site corresponds to amino acids 72 to 76. There are three main haplotypes reported so far, namely, CVMNK (chloroquine sensitive), CVIET (chloroquine resistant) and SVMNT (chloroquine resistant)^33^. Consistently, Illumina sequencing detected CVMNK type in 3D7, CVIET in K1 and Dd2 and SVMNT in 7G8. All our clinical samples resembled the 7G8 haplotype (Supplementary Table 5). When we closely looked at the MinION reads, the alignments were hard to determine at this site due to complex patterns of mismatches, deletions and insertions spanning multiple bases (Fig. 3C). For this site, we removed the reads that had unconfident SNP calls. We then remapped the MinION reads for the representative haplotypes. Without any changes to the other parameters, the refined alignment exactly detected the respective haplotypes. Independent procedures may be needed to discriminate different haplotypes, consisting of long and complex patterns of multiple mutations. We also expect that haplotypes spanning longer regions, if there are any, could also be detected by a similar method.

The eventual goal of this study is to predict possible drug resistance for each patient based on the genetic information of the parasites. We categorized the patients with respect to patterns of SNPs that have been associated with drug resistance (Table 3). For chloroquine and sulfadoxine-pyrimethamine, we found that all samples that we processed had mutations for resistance to those drugs. Namely, all the patients had the resistance-causing SNPs at *PfCRT, PfMRP1, PfMDR1, DHFR-TS* and *DHPS*. For the resistance to artemisinin, we found inconsistencies with Illumina data for the *K13* (see below for further description on *K13*). However, all the patients had SNPs in the *PfATPase6*. Nevertheless, this gene has been proved to have no correlation with artemisinin resistance^34–36^. Another exciting finding in the light of recent research is that our sequencing results suggest that all, except for one, of our clinical samples are sensitive to atovaquone. As a single agent, atovaquone gives a recrudescence rate of approximately 30%, but in combination with proguanil, atovaquone is very effective^37^, and resistance to it will not spread because atovaquone-resistant parasites cannot develop in vector mosquitoes^38^.

**Table 3:** Summary of drug resistance phenotypes of ten clinical samples, inferred from data obtained with flow cell R7.3.

Drugs	Patients									
	2-#2	2-#5	5-#1	5-#3	5-#6	5-#39	5-#40	5-#41	5-#43	5-#46
Atovaquone	−	−	−	−	−	−	−	−	−	−
Chloroquine/mefloquine	+	+	+	+	+	+	+	+	+	+
Sulfadoxine-pyrimethamine	+	+	+	+	+	+	+	+	+	+
Artemisinin	−	−	−	−	−	−	−	−	−	−

**Figure 4:**
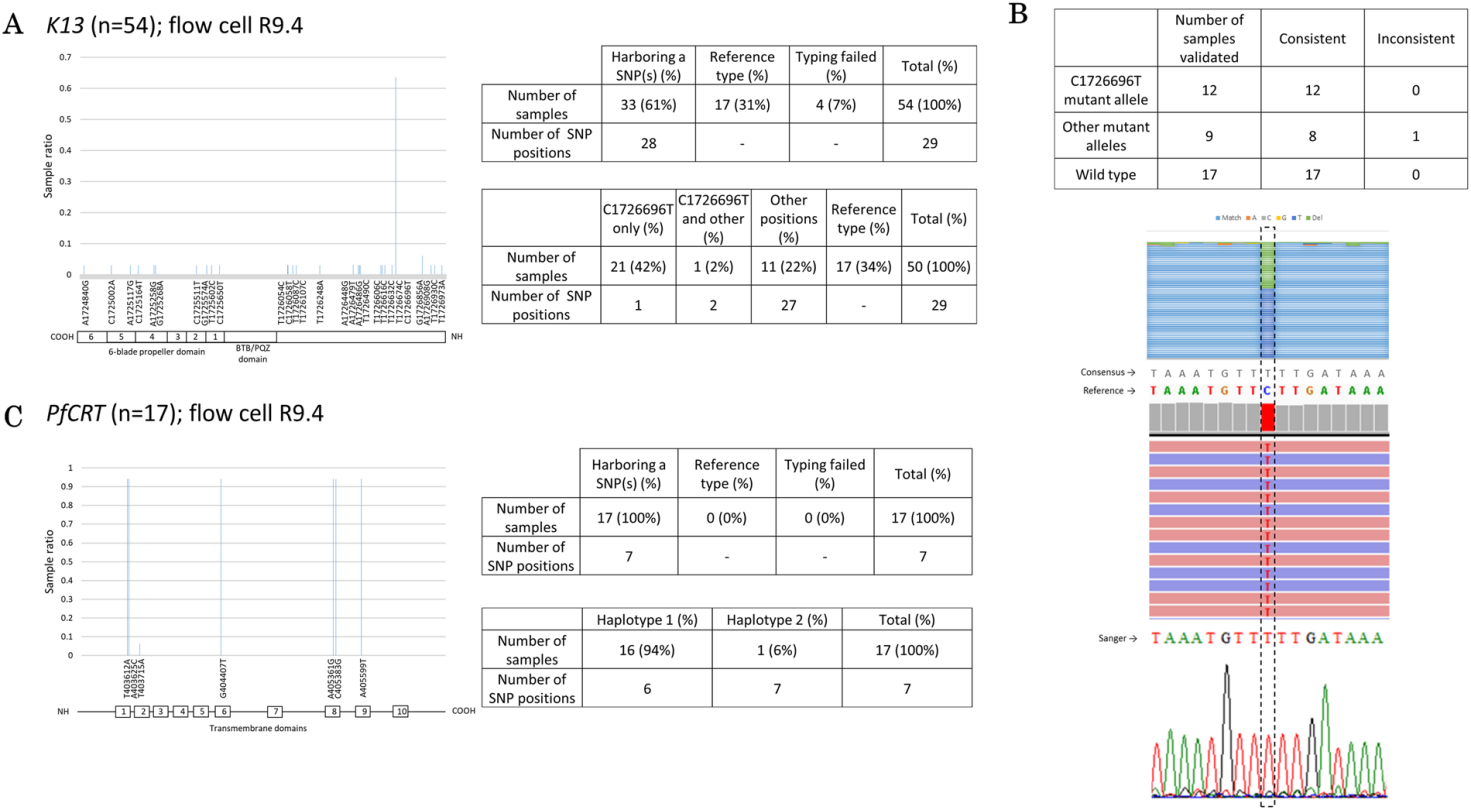
*K13* sequencing as a proof-of-concept. We sequenced the whole *K13* from 54 samples obtained in Indonesia. There are 61.1% samples with SNPs in 28 positions. A significant number of samples (42%) have SNPs in genomic position 1726696 of chromosome 13, which is in the region of *K13*. The respective K13 protein domains in regard to genomic positions is shown (**A**). The polymorphism in genomic position 1726696 is validated consistently with other sequencing means. An example of this validation with Sanger and/or Illumina is shown (**B**). Sequencing of *PfCRT* from 17 samples shows SNPs in 7 positions. We further found two haplotypes with most samples are of haplotype 1 (see Supplementary Figure 8B). The respective PfCRT protein domains in regard to genomic positions is also shown (**C**). All data are obtained from the sequencing of clinical specimens collected with FTA card and sequenced with flow cell R9.4.

We further intended to expand the dataset for the *PfCRT* and *K13* genes. This extensive analysis also should serve as a model for epidemiological study of a gene. We collected 54 parasite-positive bloods and preserved the DNA using Whatman’s FTA Elute cards, which is a common means for sample collection and storage in the field. We purified the DNA, PCR-amplified *K13* for its entire gene region, and MinION-sequenced the amplicons using flow cell R9.4. The genotyping was successful for 50 samples. We identified that 33 samples (61%) had at least one SNP at a total of 28 positions (Fig. 4A, upper right panel). The remaining 17 samples completely matched the genotype of the reference genome. The detected SNPs included a frequent C1726696T SNP which was observed in 22 samples (Fig. 4A, lower right panel; this SNP was also observed by Illumina in 3 of the initially analyzed 10 clinical samples). The other 27 SNPs were mostly observed only in one sample (“singletons”). All these mutations could be compiled into 11 mutation haplotypes (Supplementary Figure 8A). For these SNPs, we validated 20 samples using Sanger and Illumina sequencings. We found that almost all the detected SNPs were validated (Fig. 4B, upper panel). This included the high frequency SNP, which were always validated (example of validation of this SNP is shown in Fig. 4B, lower panel). Lastly, we similarly conducted the genotyping analysis of *PfCRT* gene for 17 samples (Fig. 4C). All samples had valid mutations at a total of 7 positions, converged to one major and one minor haplotypes (Fig. 4C, lower right panel). The major haplotype was mostly like that of the 7G8 strain (Supplementary Figure 8B). It is intriguing that the distinct mutation patterns between *K13* and *PfCRT* gene should be associated with the fact that the administration of chloroquine has been suspended and substituted with artemisinin for more than ten years due to the wide-spread of the drug-resistant phenotype. This data however cannot tell us whether the detected SNPs in the *K13* were caused by normal genetic drift, which is common in malaria parasite^39^, or functionally relevant SNPs invoked by the selective pressure from frequent use of artemisinin in this area. The low frequency mutation phenomenon in *K13* is also common to previous research^40^. Also, none of our finding is in agreement with the four Asian mutations that have been validated^41^. Nevertheless, the C1726696T SNP, which changes arginine to lysine at amino acid position 101 (R101K), has now dominated the parasite population in this area. Taking all the results together, we consider that genetic drifts may be emerging world-wide, which may eventually lead to a novel drug-resistant mutation. This is also in agreement with the observation that artemisinin resistance is different from other drugs, in terms that it can sporadically appear^42^.

These results are consistent with the fact that the parasite population in North Sulawesi is still susceptible to artemisinin, but resistant to chloroquine and sulfadoxine-pyrimethamine in the clinical practices. Nevertheless, one district in East Indonesia is known to have a high degree of resistance to artemisinin combination therapy (ACT), with 52% and 84% response rates for artesunate + amodiaquine and dihydroartemisinin + piperaquine, respectively^43^. The migration of the parasites from the resistant area to North Sulawesi, if happened, would have significant impact on the treatment strategy in this region as well. A vigilant surveillance is required to monitor the spread of the resistance in the surrounding regions.

### Fidelity of the generated dataset

Having collected all the datasets, we carefully re-considered the overall performance of our pipeline, especially because there are already many publications regarding SNPs found in *P. falciparum*. Therefore, using four public datasets, we compared the SNPs found in this research (from both flow cell R7.3 and R9.4) to the published datasets. PlasmoDB^44^ is a collection of 1,775,595 SNPs collected from all over the world. *P. falciparum* Community Project^45^ curates 681,587 SNPs of 3,488 samples from 23 countries. Another database is Pf3k pilot data release 4^46^, that contains 944,270 SNPs of 2,512 samples from 14 countries. Specifically for *K13*, we added a comparison with a comprehensive report by Ménard D *et al*.^41^. We found that 4 out of a total of 45 positions (excluding *K13*-propeller) were not overlapping with the datasets, suggesting the possibility of novel SNPs (Table 4). We had validated these SNPs with Illumina (Supplementary Figure 9) and found that these SNPs were not errors found by MinION. In case of *K13*, only one of our singleton SNPs in the propeller region shared a synonymous mutation with *P. falciparum* Community Project and Pf3k datasets. Any other mutations in *K13* do not intersect with the datasets. While it is true that each of the sequences produced by the MinION is still error-prone, the redundant reads of the sequences may have been able to complement the drawback. We have conducted theoretical validation regarding this issue and found that when the base is covered by >10 sequences with the quality value >10, the sequence error should be less than 0.2% (data not shown). Our novel SNPs met these criteria. We further examined the mutual overlaps of the novel SNPs in our dataset and found that 20% were common to more than one samples.

**Table 4:** A summary showing the comparison of SNPs found by this paper’s pipeline to four public databases.

Database or study name	Total SNPs	Total studies or samples	Total SNPs in database shared with this paper target genes	Total positions shared with the database	Total positions not shared with the database
PlasmoDB	1,775,395	16 studies	673	34	11
*P. falciparum* Community Project	681,587	3,488 samples from 23 countries	831	39	6
Pf3K	944,270	2,512 samples from 14 countries	884	30	15
Total positions unique to our sequencing data (excluding *K13*-propeller)					4^b^
Ménard D *et al*.^a^ (*K13*-propeller only)	108	14,037 samples from 59 countries	108	0	10
Total positions unique to our sequencing data (including *K13*-propeller)					13^c^

Data is obtained with flow cell R7.3 and R9.4.

^a^Ménard, D. *et al*. A worldwide map of Plasmodium falciparum K13-propeller polymorphisms. N. Engl. J. Med. 374, 2453–2464 (2016). ^b^One mutation in *K13* non-propeller region (R101K) is included here due to its significant presence in the clinical samples. ^c^One synonymous mutation (627A) in *K13*-propeller region is excluded because it is found in two other databases.

Also, we tested the consistency of invariant positions with the datasets. As invariant positions are considered neutral, we expect that our sequencing data should also be neutral in these positions. A low consistency would suggest that MinION introduced a lot of errors and therefore low reliability. First, we sought for non-SNP positions in the datasets which intersecting with all three datasets. We found that from a total 21,337 positions in the coding region of the target genes, 20,167 positions were non-SNPs. We then matched these positions to our sequencing data and found that 20,151 (99.9%) positions were intersecting with the non-SNPs positions (Supplementary Table 6). This result tells that MinION can show good consistency when sequencing coding regions. Taking all these results together, we conclude that these novel SNPs should be correctly representing novelty.

### Sequencing analysis in other areas

**Figure 5:**
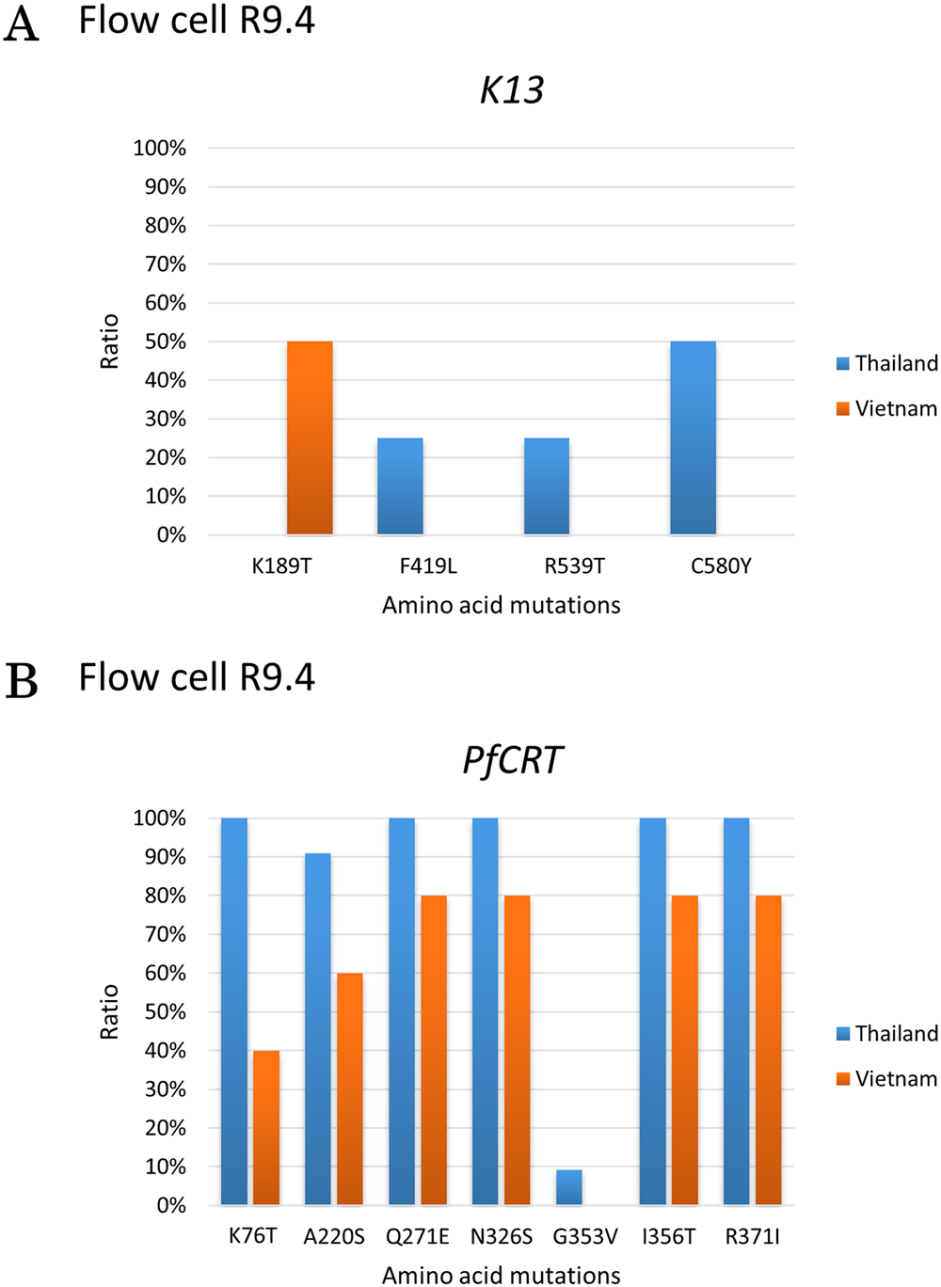
*K13* and *PfCRT* sequencing of Thailand and Vietnam samples. We sequenced 3 and 5 cases (*K13*) and 10 and 5 cases (*PfCRT*), from Thailand and Vietnam, respectively. Mutations in *K13* from Thai samples show a different pattern compared to Vietnamese samples (**A**). Nevertheless, mutation pattern of *PfCRT* is the same between Thai and Vietnamese samples (**B**). All data are obtained with flow cell R9.4.

To further enrich our data, we also sequenced *PfCRT* and *K13* of clinical samples from Thailand and Vietnam using flow cell R9.4. As expected, the samples that we acquired from Thai-Cambodia border mostly have the artemisinin-resistance associated SNPs. We found F419L, R539T, and C580Y mutations from Thai samples. On the contrary, the samples from Vietnam contains mutation in the non-propeller region (K189T) (Fig. 5A). These results are in contrast with *K13* sequence data acquired in North Sulawesi, Indonesia. Of these mutations, only R539T and C580Y that have been verified by an international consortium as artemisinin-resistance markers^15^. As for *PfCRT*, all the samples from Thailand and Vietnam has the classic markers for chloroquine resistance, i.e., K76T and A220S (Fig. 5B).

**Figure 6:**
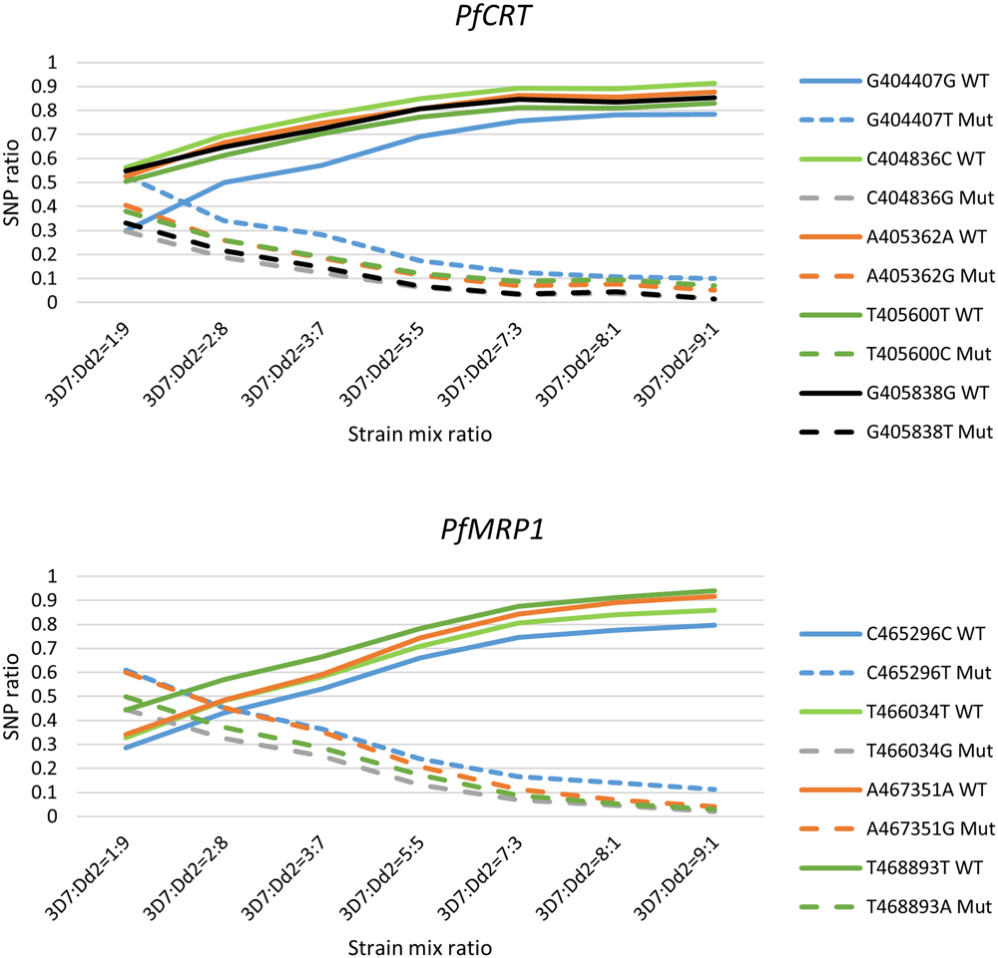
Ratio of wild type and mutated nucleotides in various proportion of a mixture of two *P. falciparum strains*, 3D7 and Dd2. A mixture of two *P. falciparum* strains (3D7 and Dd2) with different ratio show that we could infer the possibility of multiple strain infection if the SNP ratio is less than 0.5. Each color in the figure represents the position of the SNPs of *PfCRT* and *PfMRP1* inferred from Fig. 2B. Solid lines show the wild type nucleotides found in 3D7, while the dotted lines show the SNPs found in Dd2. The intersection of the solid and dotted lines is a possible mix infection, which is at the ratio of less than 0.5. Data is obtained with flow cell R9.4.

We also attempted to address how our approach can detect multiple parasite genomes in a single individual, namely complexity of infection (COI) cases^47^. Clinically, it can have an effect for the disease outcome. The results of the hitherto described analyses seemed indicating that there is possibility of multiple genome within a sample. At these positions, the base variation could not be explained by simply detecting the presence or absence of a SNP. Since we employed conservative criteria to reduce false positive calls, the called SNPs were homozygous in a given sample (all the sequences represented a variant except for possible sequencing errors). When we closely looked at the seemingly heterozygous SNPs, we detected a total of 13 of such sites. However, closer inspection at the Illumina sequencing data do not support them. Indeed, the areas in Indonesia where we collected the samples do not have malaria cases as high as those areas where mixed infections are indicated. To further address these issues, we examined to what extent multiple strain infections could be detected by our pipeline. We produced a serial mix of 3D7 and Dd2 strains. We amplified *PfCRT* and *PfMRP1* and looked for the known polymorphic sites from these genes as inferred from Fig. 2B. We called for SNPs and counted ratio of SNPs to the wild type nucleotides. As Fig. 6 shows, we theoretically could detect a possible mix infection if the ratio of a SNP is less than 0.5. We could interpolate this result to our pipeline as threshold X < 0.5. By taking into consideration multiple known SNPs and this parameter, we might detect and suspect multiple strain infection. However, at the same time, it is shown that we must be prepared for the increasing rate of the false detections, depending on the mixture rate.

## Conclusions

In this study, we described an application of the MinION sequencer for sequencing of malaria clinical samples. MinION could generate reads with long sequences and acceptable quality. Sequence accuracy was less than 90%, even with the newest flow cell. However, by compiling more than 50 sequences in depth, our in-house-developed bioinformatics pipeline achieved overall precision and recall rates of 1 and 0.97, respectively. Further improvements with new chemistry is proved to significantly increase the precision and recall while reducing the error rate. Additionally, more sophisticated methods, such as Bayesian algorithms or deep learning, could be used to construct a better analytical pipeline. A rapid examination method using portable sequencing technology will be extremely beneficial in many aspects of infectious diseases, particularly in developing countries where modern sequencing instruments are rarely available.

Our method combines PCR amplification and the portability of MinION sequencing. Coupled with PCR, it might seem impractical, but it is still can be performed in the field hospital, especially because there is a portable thermal cycler that is easy to be installed. We agree that Sanger method would be unavailable in the field, but we present Sanger method in this paper as a validation for our results. Therefore, it would not be performed routinely in this method, but as a quality control, we might employ Sanger or Illumina sequencing occasionally.

We believe these extensive analyses have strengthened our claim that the on-site sequencing is the practical approach to genotype malaria parasites, whose acquisition of the drug resistance is a substantial threat to the human health world-wide. Further, the convenient use of the sequencing method will expand our database repertoire of the parasites and will enrich our basic knowledge on its epidemiology regarding their geographical distributions and changes overtime. Towards such goal, we hope this paper paves the first step.

## Materials and Methods

### *P. falciparum* laboratory culture and clinical samples

*P. falciparum* strains 3D7, Dd2, 7G8, and K1 were used in this study. The parasite was incubated at 37 °C, 5% CO_2_ in a flask containing 10 ml of complete medium (see Supplementary Table 7 for culture recipe) with a starting parasitemia of 0.5% for 4 days. The parasite solution was then collected by 600 × *g* centrifugation at room temperature for 6 minutes. Clinical samples were obtained from 64 parasite-positive patients visiting a clinic at Sam Ratulangi University in Manado, Sulawesi, Indonesia, with informed consent and approved by the Ethical Committee of Sam Ratulangi University. Ten samples were preserved with PAXGene DNA Blood tubes (PreAnalytix) and 54 samples were preserved with FTA Elute cards (Whatman). Additional 11 and 5 parasite-positive samples were obtained from Faculty of Tropical Medicine, Mahidol University, Bangkok, Thailand and National Institute of Hygiene and Epidemiology, Hanoi, Vietnam, respectively. For Complexity of Infection (COI) experiment, we mixed laboratory culture of 3D7 and Dd2 strains with parasitemia ratio of 1:9, 2:8, 3:7, 5:5, 7:3, 8:1, and 9:1. All experiments were performed in accordance with the relevant guidelines and regulations.

### DNA extraction

We extracted culture solution and blood specimens using the DNaeasy Blood & Tissue Kit (Qiagen) according to the manufacturer’s protocol. As for DNA that was preserved with FTA Elute cards, we first eluted the DNA by submerging a part of an FTA card in 100 µl distilled water and boiling at 95 °C for 30 minutes. The subsequent extraction was similar to culture solution and blood specimens. Briefly, we added 100 µl of culture solution, EDTA-treated blood, or FTA card elute to 20 µl proteinase K and adjusted the final volume to 220 µl with PBS. We then added 200 µl of buffer AL to this solution. After incubation at 56 °C for 10 minutes, we added 200 µl of absolute ethanol. Following vortexing, we transferred all volume of the solution to a DNeasy Mini spin column attached to a 2 ml collection tube. Centrifugation at ≥6000 × *g* was performed for 1 minute. We discarded the flow-through and added 500 µl of Buffer AW1 the spin column. Centrifugation at ≥6000 × *g* was performed again for 1 minute. After discarding the flow-through, we added 500 µl of Buffer AW2 to the spin column and centrifuged it at 20,000 × *g* for 3 minutes. Elution was completed with the addition of 200 µl buffer AE to the spin column, incubation for 1 minute at room temperature, and further centrifugation at ≥6000 × *g* for 1 minute.

### Polymerase chain reaction (PCR)

We performed PCR with 1 µl of template using KAPA Hifi (Kapa Biosystems) according to the manufacturer’s protocol. PCR was executed with the following temperatures: 94 °C for 2 minutes; 30 cycles of 94 °C for 15 seconds, 42 °C for 2 minutes, 60 °C for 1 minute; and 60 °C for 2 minutes. Supplementary Figure 10A and B show the part of genes amplified and the primers used to amplify those parts. Supplementary Figure 10C and D show the agarose gel images of the amplicons. DNA was measured and assessed using a BioAnalyzer (Agilent).

### MinION library preparation

MinION sequencing libraries were prepared using the Nanopore Sequencing Kit [SQK-MAP005 or SQK-MAP006, Oxford Nanopore Technologies (ONT)] according to manufacturer’s protocol. Four lab strains re-sequencing; 54 additional samples from North Sulawesi, Indonesia; 11 samples from Mahidol University, Bangkok, Thailand; 5 samples from NIHE, Hanoi, Vietnam; and 7 mixtures of 3D7 and Dd2 strains were prepared using the 1D^2^ Sequencing of Genomic DNA Kit (SQK-LSK308, ONT). Briefly, the protocol for SQK-LSK308 is as follow: a total 1 µg of input DNA (a mixture of PCR amplicons from nine genes) was end-repaired and A-tailed using 1x Ultra II End-prep enzyme (New England Biolabs) incubated in 20 °C for 5 minutes and 65 °C for 5 minutes. Following the reaction, we purified end-prepped PCR amplicons with 1x AMPure XP (Beckman Coulter) and eluted the DNA in 25 µl nuclease-free water. A minimum of 500 ng of DNA was ligated with 1D^2^ adapter (ONT) using 1x Blunt/TA Ligase (New England Biolabs) in room temperature for 10 minutes. After purification of adapted PCR-amplicons with 1x AMPure XP (DNA was eluted in 46 µl of nuclease-free water), we ligated 400 ng of Barcode Adapter Mix (ONT) to the adapted PCR-amplicons with 1x Blunt/TA Ligase (New England Biolabs) for 10 minutes in room temperature. Purification was achieved with 140 µl of Adapter Binding Buffer (ONT) twice using the magnetic stand and the pre-sequencing mix was eluted with 15 µl of Elution Buffer (ONT). Of this, 12.5 µl of pre-sequencing mix was administered to flow cell version FLO-MIN107 in a mix of 35 µl Running Buffer with Fuel Mix (ONT) and 25.5 µl of Library Loading Buffer (ONT).

### MinION sequencing and base-calling through Metrichor

Sequencing was performed for 48 hours using flow cell version FLO-MAP003. After sequencing, base-calling (r7.X 2D Base-calling revision 1.9 or version 1.24, 2D Base-calling for SQK-MAP005 version 1.48, 2D Base-calling SQK-MAP006 version 1.62) was performed via the ONT’s Metrichor cloud-based service. As for sequencing using the new chemistry, we conducted 48 hours sequencing using flow cell version FLO-MIN107. We performed offline base-calling using ONT’s Albacore version 1.2.5 with the command line: full_1dsq_basecaller.exe–flowcell FLO-MIN107–kit SQK-LSK308–output_format fast5,fastq–input [directory of FAST5 file]–save_path [directory of the output FASTQ files] -r -t2.

### Alignment of MinION sequencing data

We used Poretools^48^ version 0.5.1 to convert FAST5 to FASTQ files for experiments base-called with Metrichor. Both “pass” and “fail” reads were used. Two aligners, BWA^49^ version 0.7.13 and LAST^28^ version 704, were used with the following parameters: BWA MEM (command line: bwa index [reference genome in FASTA file name] | bwa mem -x ont2d [indexed reference genome file name] [FASTQ file name]), LAST with default parameters (command line: lastdb [reference genome file name] [reference genome in FASTA file name] | lastal -Q1 [reference genome file name] [FASTQ file name]| last-split -m1), LAST with tuned parameters (command line: lastdb [reference genome file name] [reference genome in FASTA file name] | lastal -a12 -A15 -b3 -D1e7 -pATMAP -m100 -Q1 [reference genome file name] [FASTQ file name] | last-split -m1), and LAST-train parameters (command line: lastdb [reference genome file name] [reference genome in FASTA file name] | lastal -a11 -A14 -b3 -B3 -S1 -pATMAP -Q1 [reference genome file name] [FASTQ file name] | last-split -m1). *P. falciparum* strain 3D7 genome was used as reference genome.

### Detection of SNPs

Reads aligned to the 3D7 genome were extracted. The sequencing depth of each base was calculated, and a consensus sequence was constructed. The consensus sequence was compared with the reference sequence, and any difference was considered a SNP. The detected SNVs were validated using Illumina.

Consensus sequences were constructed as shown in Supplementary Figure 3. When the depth of a given position was less than the threshold R, where R is the depth variable, the base was considered “N”. A base (A, T, G, or C) in a given position was called if the ratio for a base to the sum of all other bases was more than the threshold X. When the base could not be determined, the position was called “n”. For heterozygous sites, a second base was called in a similar way. Variant calling using Nanopolish^32^ version 0.7.1 was performed with default parameters. We conducted these procedures with in-house scripts.

We validated our results with Illumina sequencing. A library was constructed using LT TruSeq DNA according to the manufacturer’s protocol from 1 µg of PCR amplicons. Sanger sequencing was outsourced to FASMAC Japan.

### Data access

The MinION sequencing data from the cell lines were deposited in the DNA DataBank of Japan (DDBJ) under the accession number DRA005565.

## Electronic supplementary material

Supplementary Files

Supplementary Dataset 1
