# Supplementary material for: Nanopore sequencing of drug-resistance-associated genes in malaria parasites, Plasmodium falciparum
Source: Sci Rep. 2018 May 29;8:8286. doi: 10.1038/s41598-018-26334-3 (PMC5974085; doi:10.1038/s41598-018-26334-3)
Supplement: Supplementary file 1 — Supplementary Files [file 41598_2018_26334_MOESM1_ESM.docx]

**Supplementary Files**

**Nanopore sequencing of drug-resistance-associated genes in malaria parasites, *Plasmodium falciparum***

Lucky R. Runtuwene ^1^, Josef S.B. Tuda ^2^, Arthur E. Mongan ^2^, Wojciech Makalowski ^3^, Martin C. Frith ^1,4,5^, Mallika Imwong ^6^, Suttipat Srisutham ^6^, Lan Anh Nguyen Thi ^7^, Nghia Nguyen Tuan ^7^, Yuki Eshita ^8,9^, Ryuichiro Maeda ^10^, Junya Yamagishi ^11^, and Yutaka Suzuki ^1,*^

^1^ Department of Computational Biology and Medical Sciences, Graduate School of Frontier Sciences, The University of Tokyo, 5-1-5 Kashiwanoha, Kashiwa, Chiba 277-8562, Japan

^2^ Faculty of Medicine, Sam Ratulangi University, Kampus Unsrat, Bahu Manado, 95115, Indonesia

^3^ Institute of Bioinformatics, Faculty of Medicine, University of Münster, Niels-Stensen Strasse 14, Münster 48149, Germany

^4^ Artificial Intelligence Research Center, Advanced Industrial Science and Technology, 2-3-26, Aomi, Koto-ku, Tokyo 135-0064, Japan

^5^ AIST-Waseda CBBD-OIL, 3-4-1 Ookubo, Shinjuku-ku, Tokyo 169-8555, Japan

^6^ Department of Molecular Tropical Medicine and Genetics, Faculty of Tropical Medicine, Mahidol University, 420/6 Ratchawithi Road, Thung Phaya Thai, Ratchathewi, Bangkok 10400, Thailand

^7^ National Institute of Hygiene and Epidemiology, 1 Yersin, Hanoi, 112800, Vietnam

^8^ Division of Collaboration and Education, Research Center for Zoonosis Control, Hokkaido University, North 20, West 10 Kita-ku, Sapporo, Hokkaido 001-0020, Japan

^9^ Department of Medical Entomology, Faculty of Tropical Medicine, Mahidol University, 420/6 Ratchawithi Road, Thung Phaya Thai, Ratchathewi, Bangkok 10400, Thailand

^10^ Division of Biomedical Science, Department of Basic Veterinary Medicine, Obihiro University of Agriculture and Veterinary Medicine, Nishi 2 Sen-11 Inadacho, Obihiro, Hokkaido 080-0834, Japan

^11^ Global Station for Zoonosis Control, GI-CoRE, Hokkaido University, North 20, West 10 Kita-ku, Sapporo, Hokkaido 001-0020, Japan

* Corresponding author: Yutaka Suzuki (ysuzuki@k.u-tokyo.ac.jp)

A.

| Alignment tools (parameter) | Successfully mapped reads |
| --- | --- |
| BWA-MEM  LAST (default) LAST (tuned)  LAST (trained) | 779.450  585,890  574,033  577,751 |

B.

C.

Supplementary Figure 1 | Statistics of different mapping software. Reads number (A), accuracy (B), and coverage (C). Data is from 3D7 strain sequencing using flow cell R9.4.

A.


B.

← Direction of the gene in the genome

**G T T T T T G − − T A T T A T T T A** C T T A C Reverse primer is binding to the allegedly mis-primed spot

Supplementary Figure 2 | Examples of fragmentations occurring subsequent to mapping (A). Possible mis-priming in *K13* gene; obtained with flow cell R7.3 (B).

Yes

(N_1_ + N_2_ + N_3_ + N_4_) ≥ R

1. Aligning MinION reads to *P. falciparum* genome.

2. Constructing consensus in each position of the targets.

**Low Depth** / **Unknown**

No

No

N_2_ / (N_1_ + N_2_ + N_3_ + N_4_) ≥ X

N_2_ ≥ (N_3_ + N_4_)

N_1_ / (N_1_ + N_2_ + N_3_ + N_4_) ≥ X

N_1_ ≥ (N_2_ + N_3_ + N_4_)

No

Yes

Yes

Yes

**Heterozygous N_1_/N_2_**

N_2_ / (N_1_ + N_2_ + N_3_ + N_4_) ≥ X

N_2_ ≥ (N_3_ + N_4_)

**Homozygous N_1_**

No

R: 10-500, X: 0.1-1.0

3. Calling SNP candidates by comparing with reference genome (*P. falciparum* strain 3D7).

4. Validating SNPs using Illumina sequencing.

Supplementary Figure 3 | SNP calling pipeline. N_1_, N_2_, N_3_, and N_4_ is the total number of nucleotides in a specific position in decreasing order. R is the depth variable. X is the stringency variable.


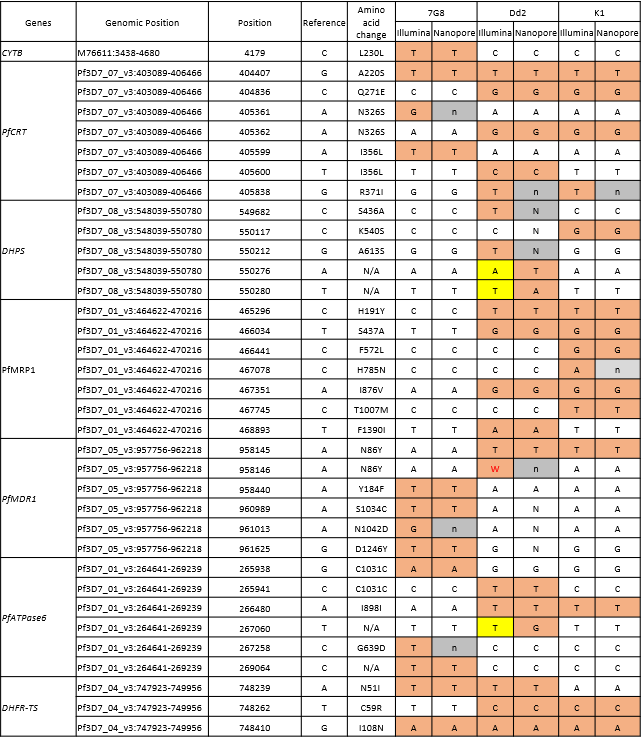
Supplementary Figure 4 | Summary of mutations found in laboratory strains. Data is obtained with flow cell R7.3.

Mutation found by Illumina or MinION

Mutation not found by Illumina

Mutation cannot be determined by MinION

Possible heterozygous SNP

W

Mutation not found by Illumina and MinION

Amino acid change is indeterminable

N/A


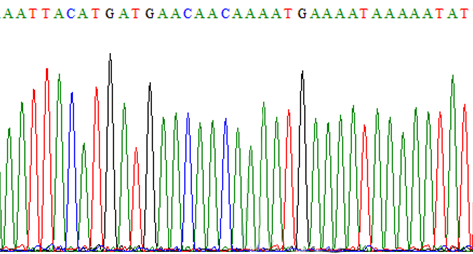

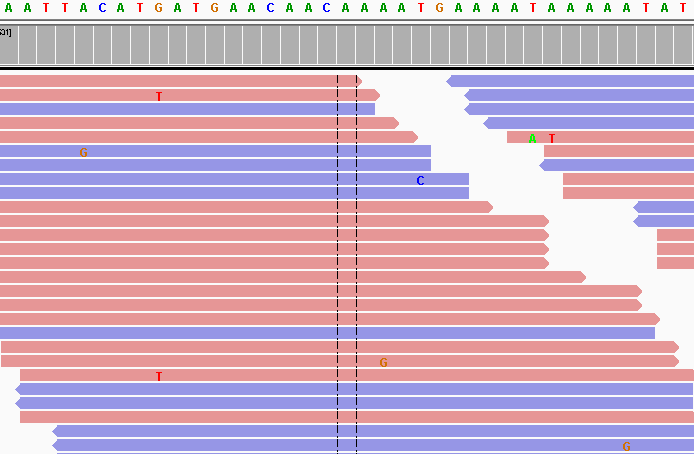
A.

Reference →

Consensus →

Sanger →

B.
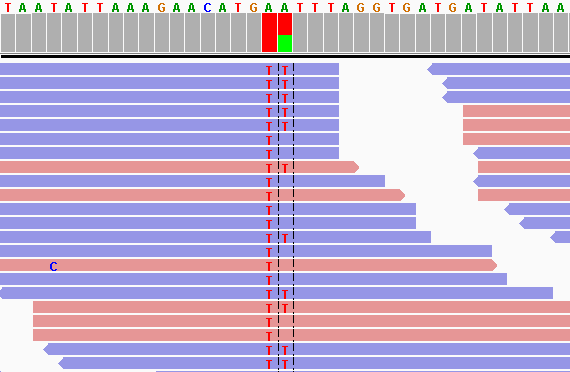

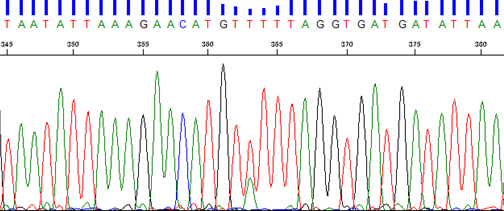


Reference →

Consensus →

Sanger →

C.
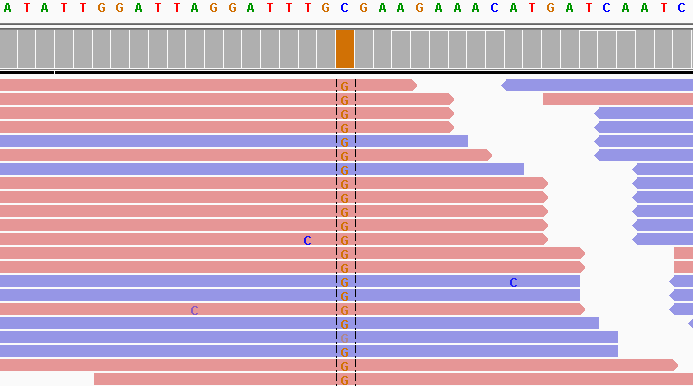

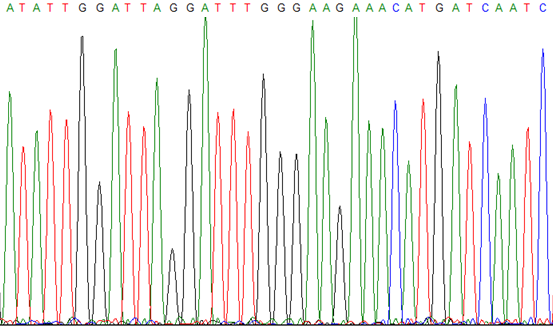


Reference →

Consensus →

Sanger →

Supplementary Figure 5 | Examples of validation to Sanger and Illumina sequencing from “false” positive (A), heterozygous SNP (B), and true positive samples (C).

Supplementary Figure 6 | The error patterns show that G to T mismatches, T deletions, and G/C insertions are the most frequent using flow cell R7.3.

MinION (R7.3)

Illumina

Nanopolish


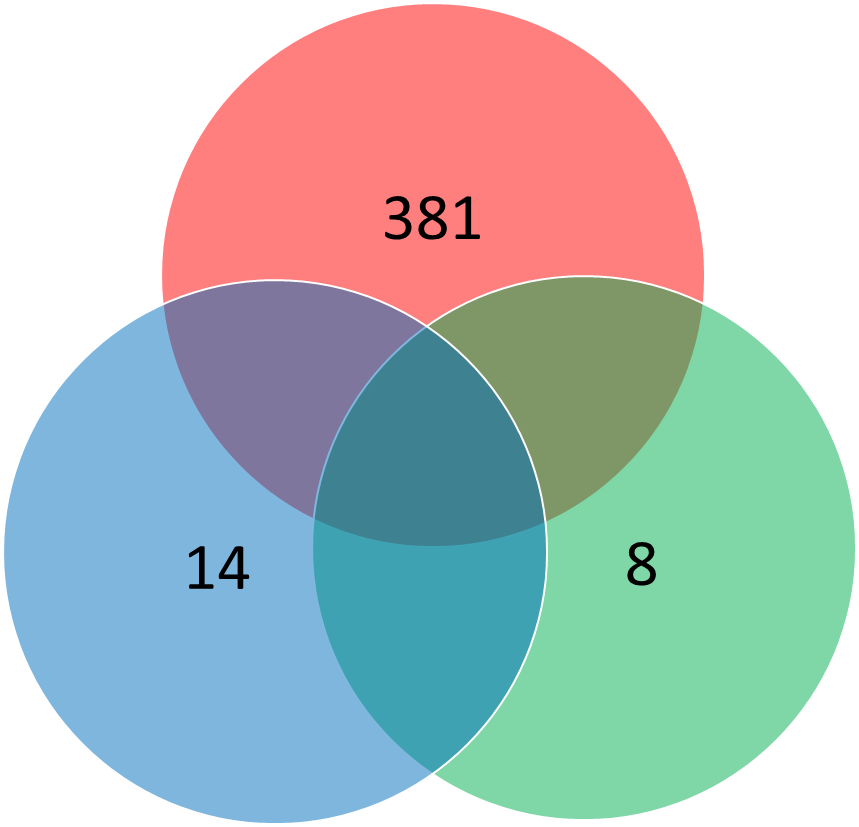


13

16

Supplementary Figure 7 | Venn diagram showing the called variants shared by three methods. MinION data were obtained from flow cell R7.3.

151

11

Genomic position

Haplotype 1

Haplotype 2

7G8

B.

A.

Haplotype 11

Haplotype 10

Haplotype 9

Haplotype 8

Haplotype 7

Haplotype 6

Haplotype 5

Haplotype 4

Haplotype 3

Haplotype 2

Haplotype 1

Supplementary Figure 8 | Haplotype of K13 (A) and PfCRT (B) from proof-of-concept experiment.

B.

A.

| Reference  Sample #5_39  Sample #5_40 | 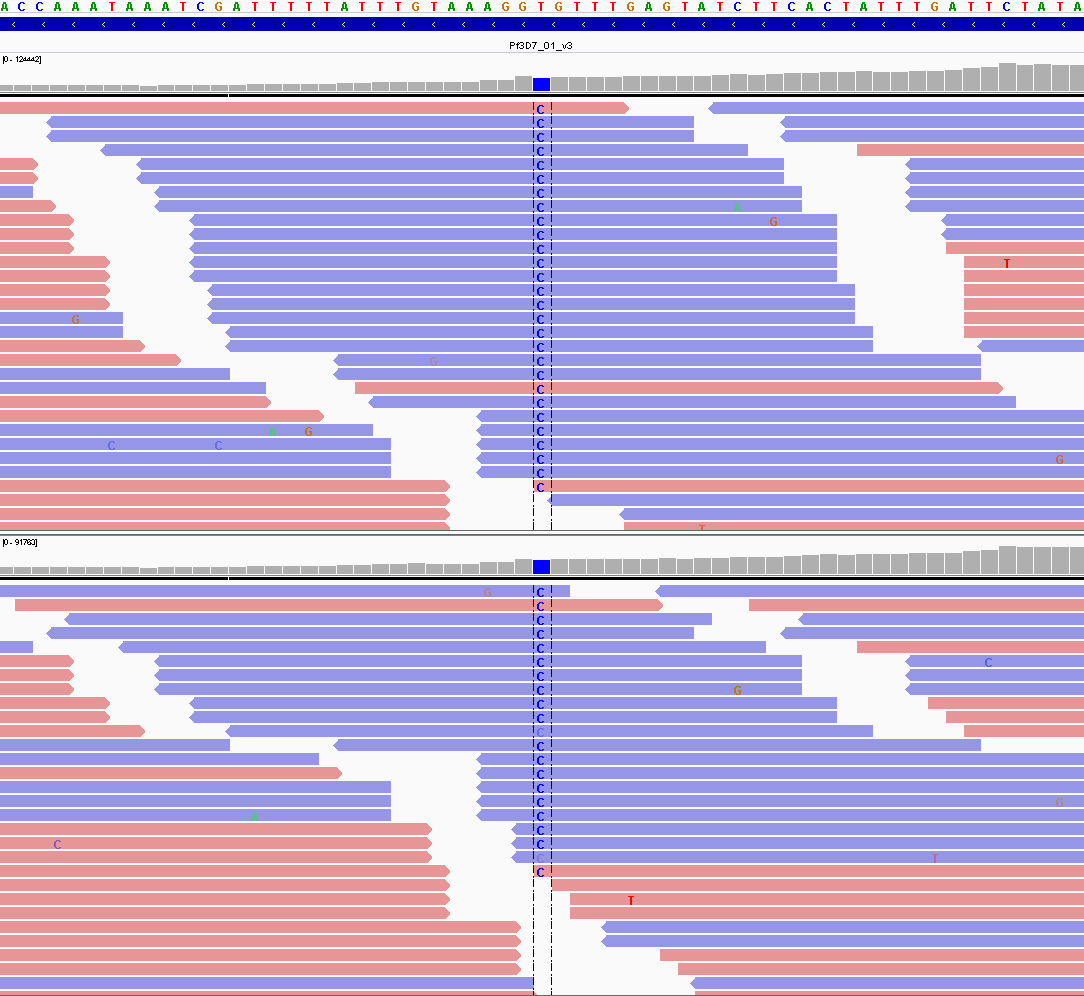 |
| --- | --- |
| Reference  Sample #5_39  Sample #5_40 | 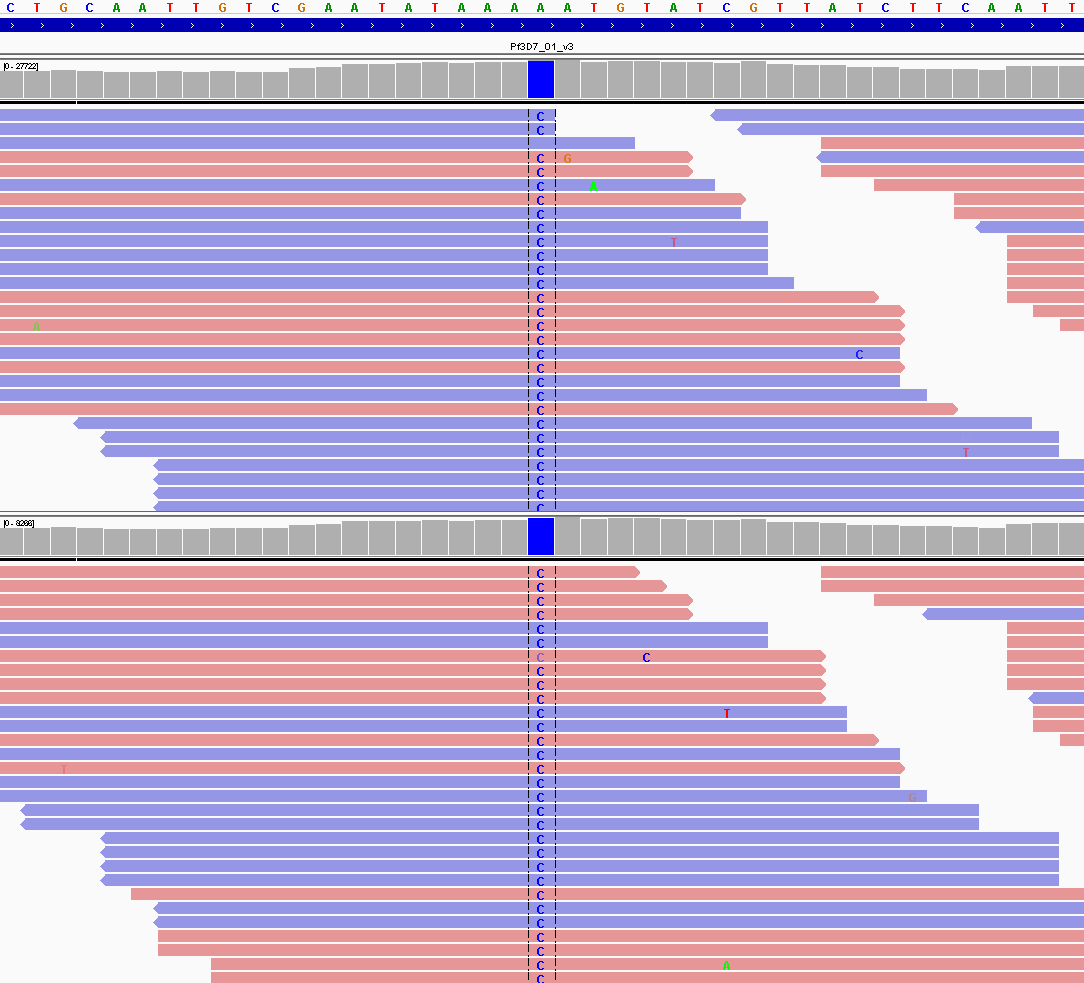 |

Supplementary Figure 9 | Illumina validation of “novel” SNPs of T to C (A) and A to C (B).

| 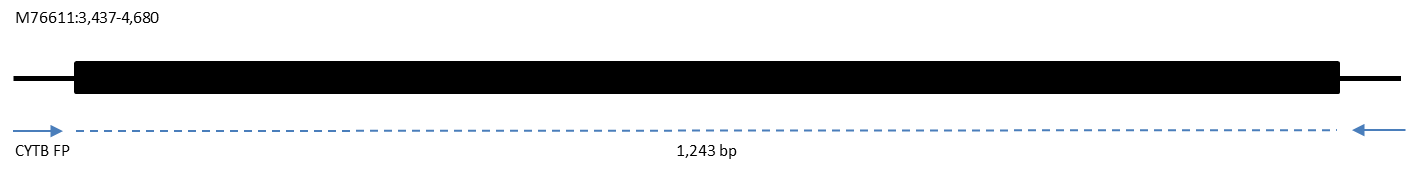A.  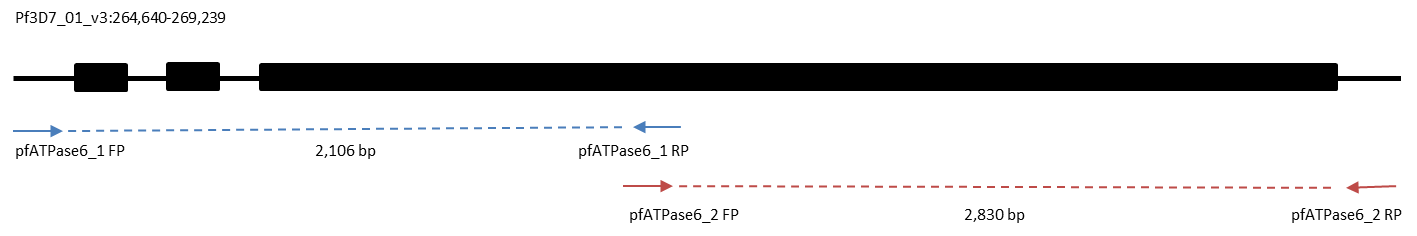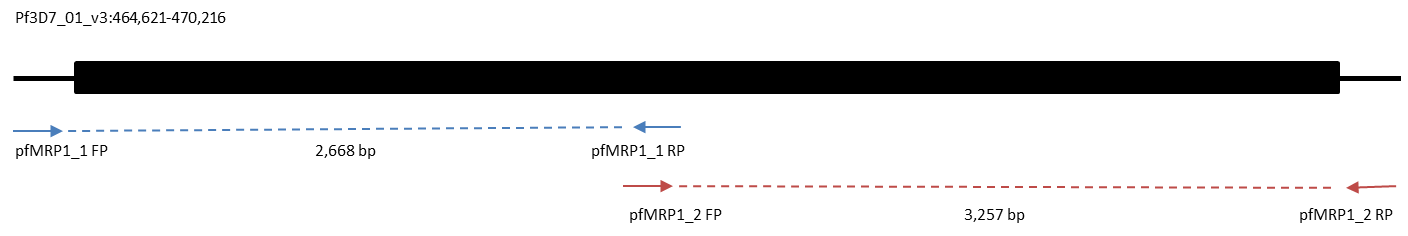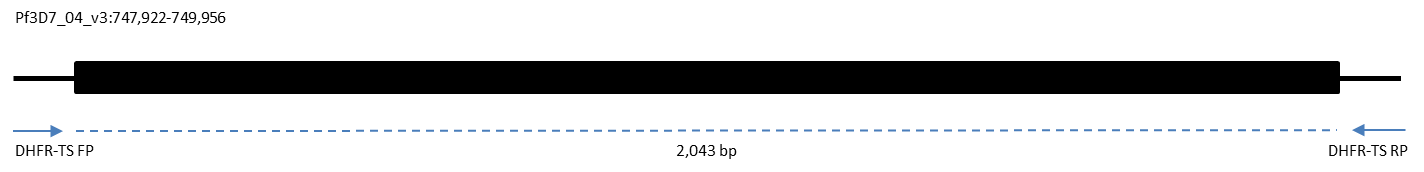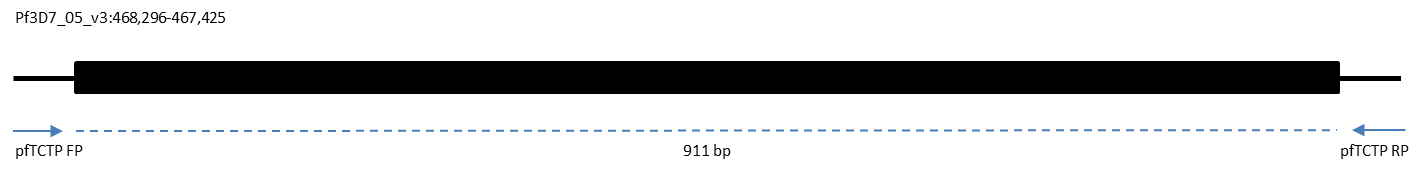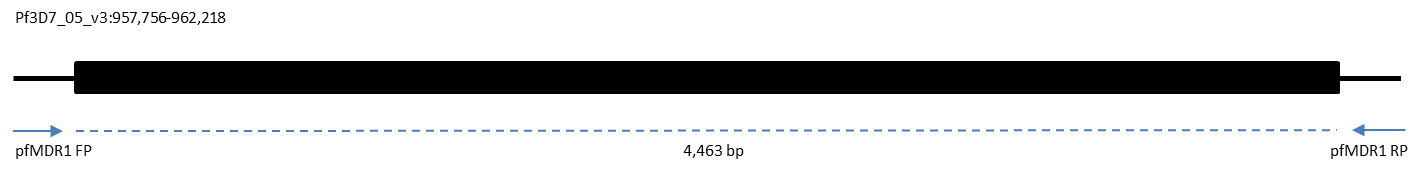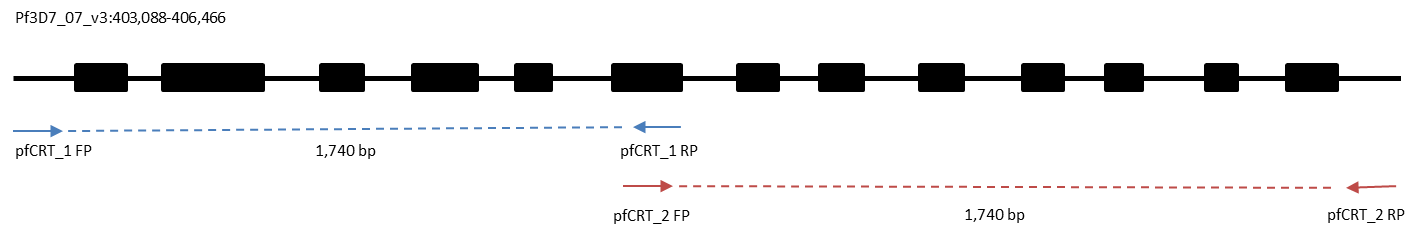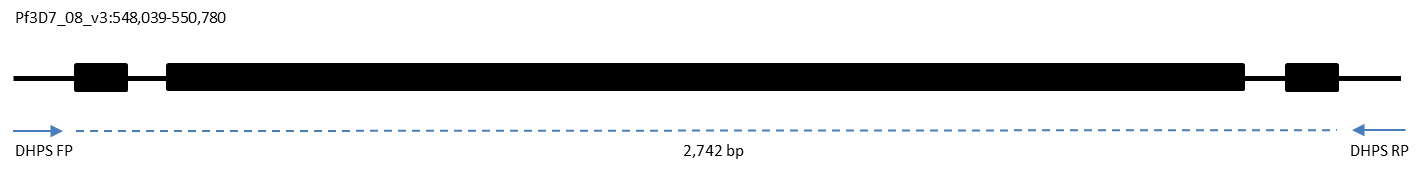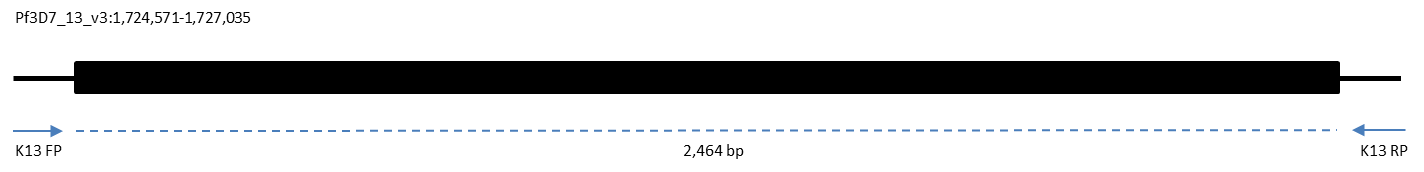 | \| Primer name \| Primer sequence \| \| --- \| --- \| \| CYTB FP \| AAT GAC TTT ATT TGG TTT AC \| \| CTYB RP \| CGG TCT GAT TTG TTC CGC TC \| \| pfATPase6_1 FP \| CTT TCA TAG GAA ATA TAA GC \| \| pfATPase6_1 RP \| AAA TAC AAC ACC TGT ACA AT \| \| pfATPase6_2 FP \| AGA AGA AAT ACA TTC ACT TG \| \| pfATPase6_2 RP \| ATT ATA TCT TTG TCA TTC GTG \| \| pfMRP1_1 FP \| TTT GGT ATG CGT ATT ACA TG \| \| pfMRP1_1 RP \| TCA TTG AAT GGA ATA TTA TAG T \| \| pfMRP1_2 FP \| GAT TTT ATT TGG AAA TGA AT \| \| pfMRP1_2 RP \| TAT TCA TGT AAT AAT ATA CCG \| \| DHFR-TS FP \| TAT TCC CAA ATA GCT AGT TC \| \| DHFR-TS RP \| AAT TTT GTC ATC ATT TGT TC \| \| pfTCTP FP \| GTA TAT AAT ATA TTG TTA GC \| \| pfTCTP RP \| TCC ATA CAT GAT ATT CAG TG \| \| pfMDR1 FP \| GTG TAC ATA GCT TAT TTC ATT TA \| \| pfMDR1 RP \| GAT CAT ATA AAT GCA TAA ATA TAA \| \| pfCRT_1 FP \| ATT TTA AAA TCG ACA TTC CG \| \| pfCRT_1 RP \| AAT GGA AGG GTG TAT ACA GG \| \| pfCRT_2 FP \| CTT ATA GGC TAT GGT ATC C \| \| pfCRT_2 RP \| TCC TTA TAA AGT GTA ATG CG \| \| DHPS FP \| AGT ATC TAT ATC TAA CTA AAA GAA A \| \| DHPS RP \| ATT AGA GTA CTT GAC ATA TAA TGA \| \| K13 FP \| GAA AAT CAT AAA CAA TCA AG \| \| K13 RP \| CAT TCA TTT ATT ATG TTT TTG \| |
| --- | --- | --- | --- | --- | --- | --- | --- | --- | --- | --- | --- | --- | --- | --- | --- | --- | --- | --- | --- | --- | --- | --- | --- | --- | --- | --- | --- | --- | --- | --- | --- | --- | --- | --- | --- | --- | --- | --- | --- | --- | --- | --- | --- | --- | --- | --- | --- | --- | --- | --- | --- |

B.


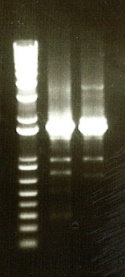

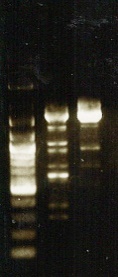

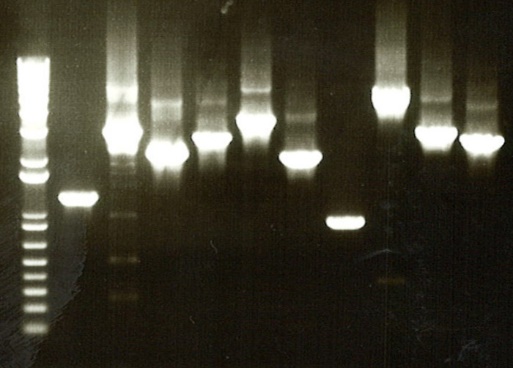

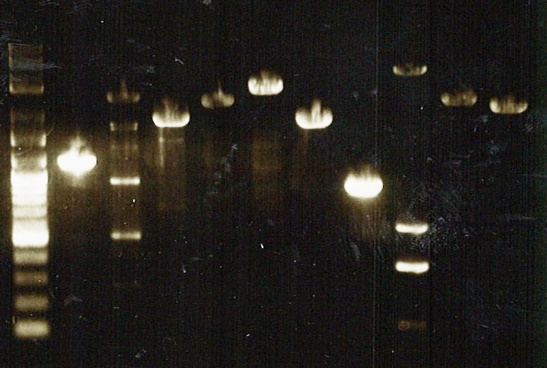
C D.

500

1,500

CRT_1 CRT_2

3D7

CRT_1 CRT_2

K1

500

1,500

500

1,000

1,500

3,000

Apo ATP6_1 ATP6_2 MRP_1 MRP_2 DHFR TCTP MDR1 DHPS K13

K1

500

1,000

1,500

3,000

Apo ATP6_1 ATP6_2 MRP_1 MRP_2 DHFR TCTP MDR1 DHPS K13

3D7

Supplementary Figure 10 | Amplified section of the genes (A) and primers used for amplification (B). Gel images from of *pfCRT* (C) and remaining eight genes (D). Each gel image was acquired from single gel.

| Genes | Drug resistance | Documented mutations |
| --- | --- | --- |
| *CYTB* | Atovaquone | M133I^1^, P275T^1^, K272R^1^, G280D^1^, L283I^1^, V284K^1^, Y268S^1^ |
| *PfATPase6* | Artemisinin | I89T^2^, E431K^3^, A623E^3^, S679N^3^, N683K^2^, I898I^3^ |
| *PfMRP1* | Chloroquine, mefloquine, quinine, artemisinin | H191Y^4^, S437A^4^, I876V^4^, F1390I^5^, K1466R^6^ |
| *DHFR* | Sulfadoxine-pyrimethamine | A16V^7^, C50R^7^, N51I^8^, C59R^8^, S108N/T^7^, I164L^7^ |
| *TCTP* | Artemisinin | No documented mutations |
| *PfCRT* | Chloroquine, mefloquine, quinine | C72S^9^, M74I^10^, N75E^10^, K76T^11^, A220S^11^, Q271E^11^, L272F^11^, N326S^11^, T333S^12^, I356L^13^, R371I^11^ |
| *PfMDR1* | Chloroquine, mefloquine, quinine, sulfadoxine-pyrimethamine | N86Y^14^, Y184F^14^, S1034C^15^, N1042D^15^, F1226Y^16^, D1246Y^15^ |
| *DHPS* | Sulfadoxine-pyrimethamine | S436A/F/C^7^, A437G^7^, K540S^7^, A581G^7^, I588F^17^, A613S/T^7^ |
| *K13* | Artemisinin | M476I^18^, Y439H^18^, R539T^18^, A578S^18^, C580Y^18^ |

Supplementary Table 1 | Summary of documented mutations among genes that were found to have a correlation with antimalarial resistance.

| Sample | Total reads (2D) | Reads that are mapped to target (LAST 581, MC Frith et al. 2010 BMC Bioinformatics) | Ratio of mapped reads |
| --- | --- | --- | --- |
| 3D7 | 43,967 | 22,646 | 51.50% |
| K1 | 35,553 | 22,018 | 61.93% |
| 7G8 | 71,621 | 39,826 | 55.60% |
| Dd2 | 28,303 | 19,339 | 68.33% |
| Total | 179,444 | 103,825 | 57.86% |

Supplementary Table 2 | Sequencing and mapping statistics for four laboratory strains of P. falciparum. Data is obtained with flow cell R7.3.

| Sample | Total reads (2D) | Mapped reads | Ratio of mapped reads |
| --- | --- | --- | --- |
| 5-#4 | 671,556 | 616,839 | 91.85% |
| 5-#24 | 815,034 | 673,664 | 82.65% |
| 5-#47 | 1,728,550 | 1,549,463 | 89.64% |
| 6-#58 | 1,607,589 | 1,137,662 | 70.77% |
| 6-#59 | 2,132,118 | 1,941,734 | 91.07% |
| 6-#64 | 1,960,066 | 1,756,455 | 89.61% |
| 6-#65 | 491,323 | 402,653 | 81.95% |
| 6-#66 | 1,339,553 | 1,094,799 | 81.73% |
| 6-#67 | 1,539,582 | 1,389,504 | 90.25% |
| 6-#76 | 983,362 | 867,911 | 88.26% |

Supplementary Table 3 | Sequencing and mapping statistics of ten clinical samples obtained with flow cell R9.4. We used new clinical samples for sequencing with flow cell R9.4 because we have exhausted the original ten samples on first analysis.


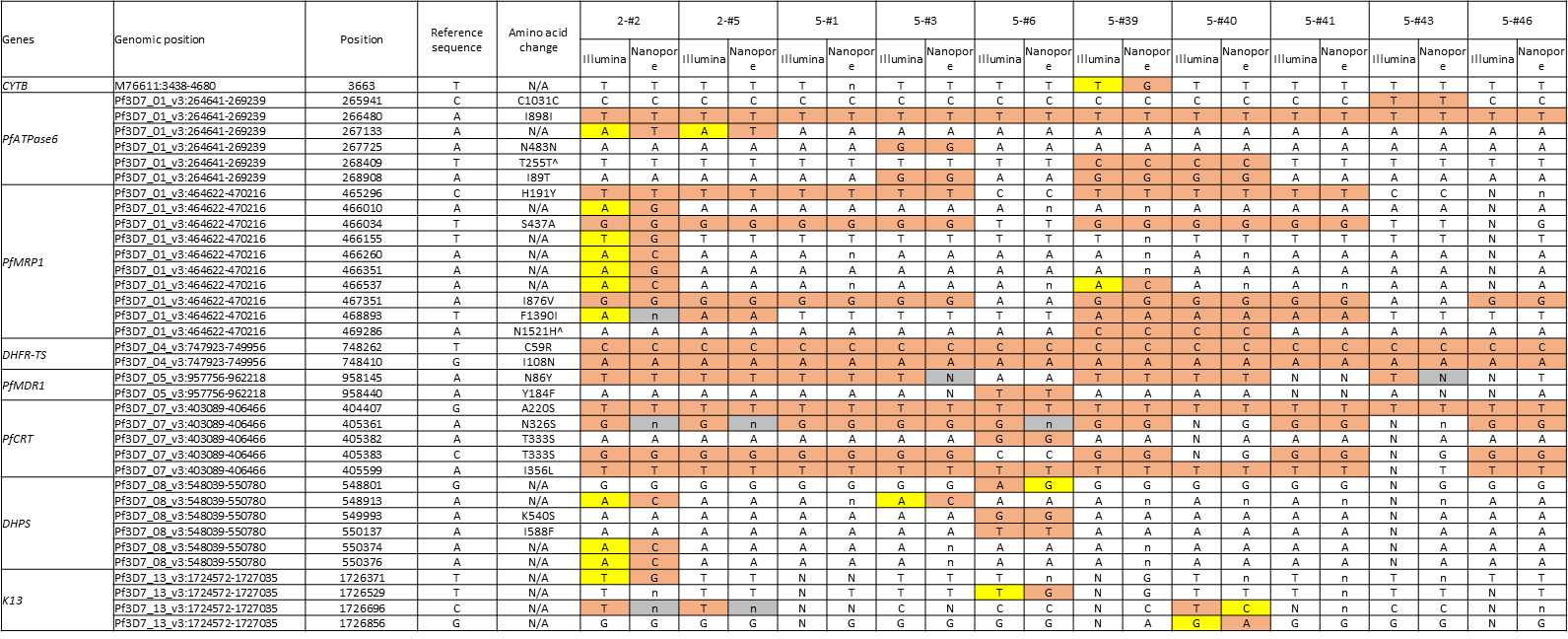


^

Mutation not found by Illumina and MinION

Mutation cannot be determined

Mutation not found by either Illumina or MinION

Mutation found by Illumina or MinION

Amino acid change is indeterminable

Previously undocumented mutation

N/A

Supplementary Table 4 | Summary of mutations found in ten clinical samples. Data is obtained with flow cell R7.3. We cannot present data from flow cell R9.4 for this analysis because we have exhausted these samples in this analysis.


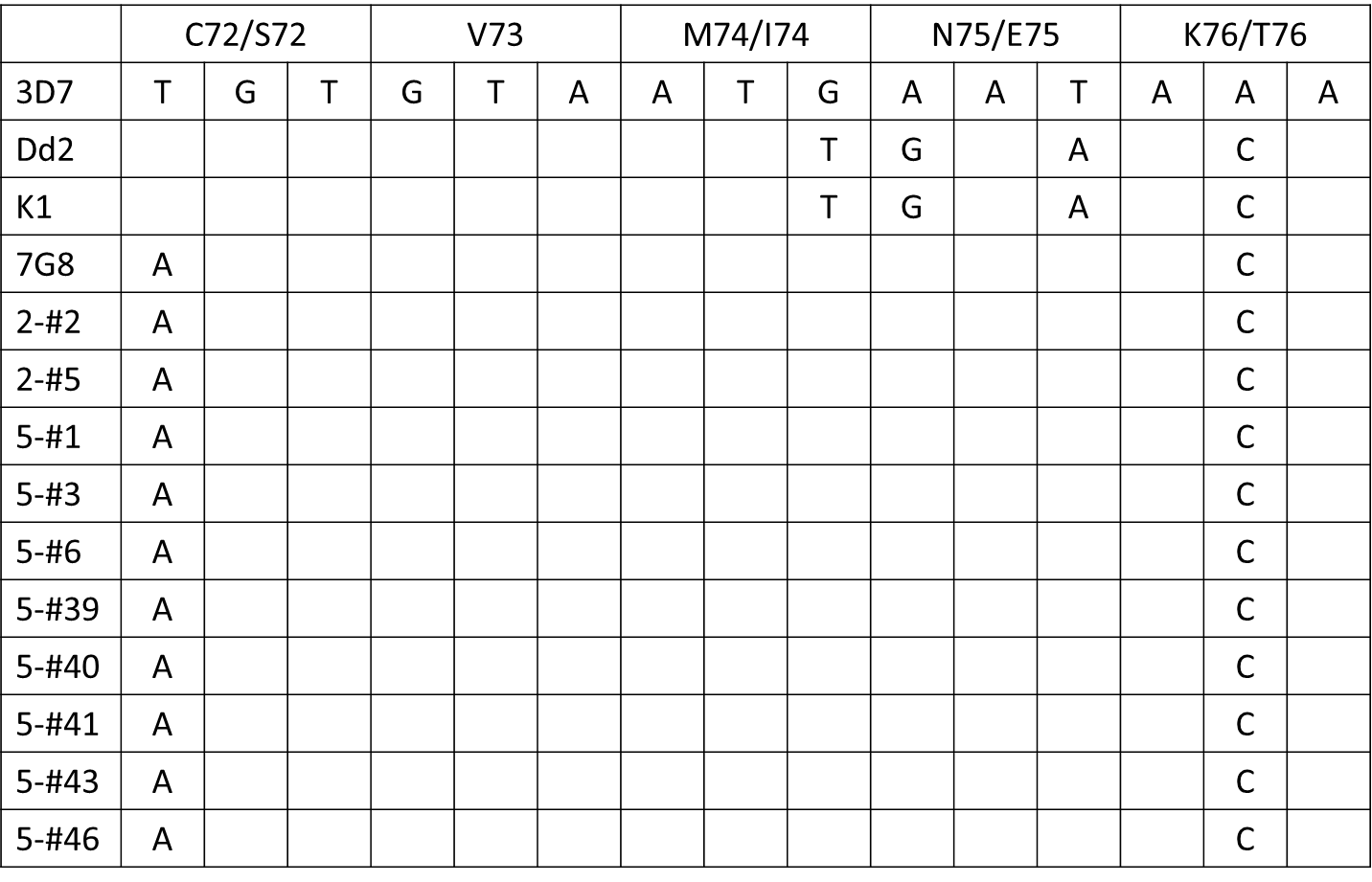


Supplementary Table 5 | Amino acids 72-76 of clinical samples’ *pfCRT* gene show resemblance to 7G8 strain. Data is obtained with flow cell R7.3.

| Gene name | Total coding region positions | Total invariant positions in coding region | Total invariant positions intersecting with public datasets | Ratio of invariant positions intersecting with public datasets |
| --- | --- | --- | --- | --- |
| *PfATPase6* | 3,687 | 3,372 | 3,369 | 99.9% |
| *PfMRP1* | 5,469 | 5,319 | 5,313 | 99.9% |
| *DHFR* | 1,827 | 1,796 | 1,796 | 100.0% |
| *TCTP* | 516 | 496 | 496 | 100.0% |
| *PfMDR1* | 4,261 | 3,935 | 3,935 | 100.0% |
| *PfCRT* | 1,275 | 1,186 | 1,186 | 100.0% |
| *DHPS* | 2,121 | 2,066 | 2,061 | 99.8% |
| *K13* | 2,181 | 1,997 | 1,995 | 99.9% |
| Total | 21,337 | 20,167 | 20,151 | 99.9% |

Supplementary Table 6 | Composition of invariant positions by gene. Data is obtained with flow cell R7.3 and R9.4.

| RPMI Basal Medium | | | |
| --- | --- | --- | --- |
|  | RPMI 1640 (Gibco #11875-093) | 500 ml |  |
|  | HEPES 1M pH7.4 | 12.5 ml |  |
|  | Gentamicin 10mg/ul (Sigma #G1272-10ml) | 200 µl |  |
| RPMI Gpf Medium | | | |
|  | RPMI Basal Medium | 512.7 ml |  |
|  | Daigo GF21 (Wako #634-25725) | 50 ml |  |
| RPMI Complete Medium | | | |
|  | RPMI Gpf Medium | 100 ml |  |
|  | Hypoxanthine (10mM) (Sigma #H9377-5G) | 1.5 ml | Use fresh every time |

Supplementary Table 7 | The recipe of malaria parasite culture.

References

1. Korsinczky, M. *et al.* Mutations in Plasmodium falciparum cytochrome b that are associated with atovaquone resistance are located at a putative drug-binding site. *Antimicrob. Agents Chemother.* **44,** 2100–2108 (2000).

2. Bertaux, L., Quang, L. H., Sinou, V., Thanh, N. X. & Parzy, D. New PfATP6 Mutations Found in Plasmodium falciparum Isolates from Vietnam. *Antimicrob. Agents Chemother.* **53**, 4570–4571 (2009).

3. Zakeri, S. *et al*. Molecular assessment of atpase6 mutations associated with artemisinin resistance among unexposed and exposed Plasmodium falciparum clinical isolates to artemisinin-based combination therapy. *Malar. J.* **11**, 373 (2012).

4. Dahlström, S. *et al*. Plasmodium falciparum multidrug resistance protein 1 and artemisinin-based combination therapy in Africa. *J. Infect. Dis.* **200**, 1456–64 (2009).

5. Gupta, B. *et al*. Plasmodium falciparum multidrug resistance protein 1 (pfmrp1) gene and its association with in vitro drug susceptibility of parasite isolates from north-east Myanmar. *J. Antimicrob. Chemother.* **69**, 2110–2117 (2014).

6. Dahlström, S., Veiga, M. I., Mårtensson, A., Björkman, A. & Gil, J. P. Polymorphism in Pfmrp1 (Plasmodium falciparum multidrug resistance protein 1) amino acid 1466 associated with resistance to sulfadoxine-pyrimethamine treatment. *Antimicrob. Agents Chemother.* **53**, 2553–2556 (2009).

7. Abdul-Ghani, R., Farag, H. F. & Allam, A. F. Sulfadoxine-pyrimethamine resistance in Plasmodium falciparum: A zoomed image at the molecular level within a geographic context. *Acta Trop.* **125**, 163–190 (2013).

8. Kamchonwongpaisan, S. *et al*. Inhibitors of multiple mutants of Plasmodium falciparum dihydrofolate reductase and their antimalarial activities. *J. Med. Chem.* **47**, 673–80 (2004).

9. Valderramos, S. G. *et al*. Identification of a mutant PfCRT-mediated chloroquine tolerance phenotype in Plasmodium falciparum. *PLoS Pathog.* **6**, 1–14 (2010).

10. Cooper, R. *et al*. Mutations in transmembrane domains 1, 4 and 9 of the Plasmodium falciparum chloroquine resistance transporter alter susceptibility to chloroquine, quinine and quinidine. *Mol. Microbiol*. **63**, 270–82 (2007).

11. Awasthi, G. & Das, A. Genetics of chloroquine-resistant malaria: A haplotypic view. *Mem. Inst. Oswaldo Cruz* **108**, 947–961 (2013).

12. Callaghan, P. S., Hassett, M. R. & Roepe, P. D. Functional Comparison of 45 Naturally Occurring Isoforms of the Plasmodium falciparum Chloroquine Resistance Transporter (PfCRT). *Biochemistry* **54**, 5083–5094 (2015).

13. Wellems, T. E. & Plowe, C. V. Chloroquine-Resistant Malaria*. J. Infect. Dis*. **184**, 770–776 (2001).

14. Veiga, M. I. *et al*. Globally prevalent PfMDR1 mutations modulate Plasmodium falciparum susceptibility to artemisinin-based combination therapies. *Nat. Commun*. **7**, 1–12 (2016).

15. Ibraheem, Z. O., Abd Majid, R., Noor, S. M., Sedik, H. M. & Basir, R. Role of different Pfcrt and Pfmdr-1 mutations in conferring resistance to antimalaria drugs in Plasmodium falciparum*. Malar. Res. Treat.* **2014**, 1–17 (2014).

16. Pirahmadi, S., Zakeri, S., Afsharpad, M. & Djadid, N. D. Mutation analysis in pfmdr1 and pfmrp1 as potential candidate genes for artemisinin resistance in Plasmodium falciparum clinical isolates 4 years after implementation of artemisinin combination therapy in Iran. *Infect. Genet. Evol*. **14**, 327–334 (2013).

17. Basuki, S. *et al*. Two novel mutations of pfdhps K540T and I588F, affecting sulphadoxine-pyrimethamine-resistant response in uncomplicated falciparum malaria at Banjar district, South Kalimantan Province, Indonesia. *Malar. J.* **13**, 135 (2014).

18. Straimer, J. *et al*. K13-propeller mutations confer artemisinin resistance in Plasmodium falciparum clinical isolates. *Science* **347**, 428–31 (2014).
